# Reversible nanocluster structure transformation between face-centered cubic and icosahedral isomers†
†Electronic supplementary information (ESI) available: Fig. S1–S23 showing the total structure, TGA and XPS results of **Pt_1_Ag_28_-2**; the stability test; DPV results; catalytic activity results; TEM images; structural comparison between **Pt_1_Ag_28_-1** and **Pt_1_Ag_28_-2**; time-dependent UV-vis spectra, PL spectra and ESI-MS results from **Pt_1_Ag_28_-2** to **Pt_1_Ag_28_-1**; detailed XAFS results; TDDFT results; temperature-dependent PL and UV-vis spectra of **Pt_1_Ag_28_-2**; the crystal data and structure refinement for **Pt_1_Ag_28_-2**. CCDC 1840953. For ESI and crystallographic data in CIF or other electronic format see DOI: 10.1039/c9sc02667c


**DOI:** 10.1039/c9sc02667c

**Published:** 2019-08-05

**Authors:** Xi Kang, Li Huang, Wei Liu, Lin Xiong, Yong Pei, Zhihu Sun, Shuxin Wang, Shiqiang Wei, Manzhou Zhu

**Affiliations:** a Department of Chemistry and Center for Atomic Engineering of Advanced Materials , Anhui Province Key Laboratory of Chemistry for Inorganic/Organic Hybrid Functionalized Materials , Anhui University , Hefei , Anhui 230601 , China . Email: ixing@ahu.edu.cn ; Email: zmz@ahu.edu.cn; b National Synchrotron Radiation Laboratory , University of Science and Technology of China , Hefei , Anhui 230029 , China . Email: zhsun@ustc.edu.cn; c Department of Chemistry , Key Laboratory of Environmentally Friendly Chemistry and Applications of Ministry of Education , Xiangtan University , Xiangtan , Hunan 411105 , China

## Abstract

Structural transformations between isomers of nanoclusters provide a platform to tune their properties and understand the fundamental science due to their intimate structure–property correlation. Herein, we demonstrate a reversible transformation between the face-centered cubic (FCC) and icosahedral isomers of Pt_1_Ag_28_ nanoclusters accomplished in the ligand-exchange processes. Ligand-exchange of 1-adamantanethiolate protected Pt_1_Ag_28_ by cyclohexanethiolate could transform the FCC kernel to the icosahedral isomer. Interestingly, the icosahedral Pt_1_Ag_28_ could be reversibly transformed to the FCC configuration when the cyclohexanethiolate ligand is replaced again by 1-adamantanethiolate. A combination of UV-vis absorption, mass spectrometry, photo-luminescence and X-ray absorption fine structure unambiguously identifies that the FCC-to-icosahedral structure transformation of Pt_1_Ag_28_ involves two distinct stages: (i) ligand-exchange induced outmost motif transformation and (ii) abrupt innermost kernel transformation. As a result of this structural transformation, the emission wavelength of Pt_1_Ag_28_ red-shifts from 672 to 720 nm, and the HOMO–LUMO energy gap reduces from 1.86 to 1.74 eV. This work presents the first example of nanocluster isomers with inter-switching configurations, and will provide new insights into manipulating the properties of nanoclusters through controllably tuning their structures.

## Abstract 2

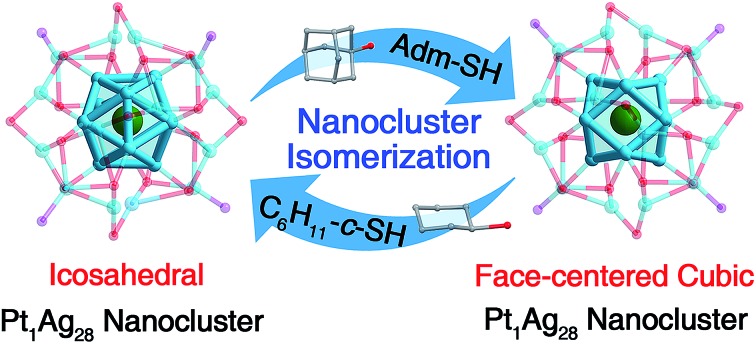
The reversible transformation between a FCC and icosahedral configuration has been achieved at the atomic level, based on Pt_1_Ag_28_ nanocluster isomers.

## 1 Introduction

Nanoclusters, with the advantages of precise compositions and well-defined structures, provide an exciting opportunity to grasp the structure–property correlation at the atomic level.^1^,^2^ The quantum size effect of nanoclusters endows them with a plethora of properties, such as photo-luminescence (PL), catalysis, chirality, and magnetism, to name a few.^1^,^2^ The property manipulation at the atomic level has long been a hot topic, and has allowed a series of nanoclusters with controllable chemical–physical properties to be produced.1f–h,2g,i,k,l

Isomerism is being intensely pursued in nanoscience, and has been exploited for tailoring the performances of nanoparticles.^3^ However, understanding the isomerism phenomenon at the atomic level has been largely impeded due to the poly-disperse sizes as well as the uncertain surface coordination modes of nanoparticles.1a,b,3b The precise structures of nanoclusters make it possible to fully grasp the isomerism phenomenon.^4^–^6^ Although stereoisomerism has been extensively studied in the nanocluster field (that is, obtaining chiral–optical nanoclusters *via* the separation of left- and right-handed isomers),^4^,^5^ structural isomerism remains rare. Up to now, only a few structural isomers in the nanocluster range have been observed (*e.g.*, Au_28_(SR)_20_, Au_38_(SR)_24_, and Au_52_(SR)_32_).6a,c–f Different structure-dependent properties (*e.g.*, optical absorption, catalytic activity, and thermal stability) have been investigated on the basis of these nanocluster isomers.5f,^6^ In addition, experimental and theoretical efforts in exploring the isomerism effect on atomic arrangement and properties of nanoclusters should be continued because such findings enable us to fully grasp the structure–property correlation, and thus help us to design new nanoclusters with unique functions.5f,^6^

Meanwhile, accompanied by the structure determination of nanoclusters resolved by single-crystal X-ray crystallography (SCXC), several efforts have been made for grasping the size-growth and structure-transformation modes from small complexes to nanoclusters, then to large nanoparticles.1l,n,^7^ Icosahedron and face-centered cubic (FCC) are the two most common configurations of nanocluster kernels. The structural transformation from an icosahedral to FCC configuration or its reverse process has been reported recently in the nanocluster field.6d,^8^ For instance, ligand-exchanging Au_25_(S-C_2_H_4_Ph)_18_ with excess ^*t*^BuPh-SH changes the icosahedral configuration of the Au_13_ kernel into a FCC configuration.6*d* Besides, the existence of Me_2_PhSH can transform Au_44_(S-Ph^*t*^Bu)_28_ into Au_44_(S-PhMe_2_)_26_, where the kernel changes from the FCC to the icosahedral configuration.8*a* However, for nanocluster isomer systems, to the best of our knowledge, the structural transformation from the FCC to icosahedron or its reverse process, not to mention the reversible transformation between these two configurations, remains incomplete. Such a shortage impedes the full understanding of nanocluster isomerism in terms of structure-transformation modes and structure–property correlation.

In the current work, a reversible transformation has been accomplished between FCC and icosahedral isomers of Pt_1_Ag_28_ nanoclusters. The ligand-exchange method is herein exploited for fulfilling the reversible transformation between Pt_1_Ag_28_(S-Adm)_18_(PPh_3_)_4_ (**Pt_1_Ag_28_-1**, where S-Adm represents 1-adamantanethiol) with a FCC Pt_1_Ag_12_ kernel and Pt_1_Ag_28_(S-*c*-C_6_H_11_)_18_(PPh_3_)_4_ (**Pt_1_Ag_28_-2**, where S-*c*-C_6_H_11_ represents cyclohexanethiol) with an icosahedral Pt_1_Ag_12_ kernel. ESI-MS, PL and EXAFS results are combined to demonstrate that the configurational transformation between the FCC and the icosahedron contains two distinct stages: the motif transformation process and kernel transformation process, where the latter transformation is induced by the former one. Accompanied by the structural transformation (from **Pt_1_Ag_28_-1** to **Pt_1_Ag_28_-2**), the emission wavelength red-shifts from 672 nm to 720 nm, and the HOMO–LUMO energy gap reduces from 1.86 eV to 1.74 eV.

## 2 Experimental methods

### 2.1 Materials

All chemicals including silver nitrate (AgNO_3_, 99.9%, metal basis), hexachloroplatinic(iv) acid (H_2_PtCl_6_·6H_2_O, 99.9% metal basis), triphenylphosphine (PPh_3_, 99%), 1-adamantanethiol (Adm-SH, C_10_H_15_SH, 99%), cyclohexanethiol (C_6_H_11_-*c*-SH, 99%), sodium borohydride (NaBH_4_, 99.9%), styrene (C_6_H_5_–C_2_H_3_, 99.5%), potassium carbonate (K_2_CO_3_, 99.5%), methylene chloride (CH_2_Cl_2_, HPLC grade), methanol (CH_3_OH, HPLC), acetic ether (CH_3_COOC_2_H_5_, HPLC), ether ((C_2_H_5_)_2_O, HPLC), and toluene (C_6_H_5_–CH_3_, HPLC) were purchased from Sigma-Aldrich and used without further purification. All glassware were thoroughly cleaned with aqua regia (HCl : HNO_3_ = 3 : 1 v/v), rinsed with copious pure water, and then dried in an oven prior to use.

### 2.2 Synthesis of **Pt_1_Ag_28_-1** nanoclusters

The synthesis of **Pt_1_Ag_28_-1** nanoclusters was carried out with reference to our previous work.8*b*

### 2.3 Converting **Pt_1_Ag_28_-1** into **Pt_1_Ag_28_-2** nanoclusters

Specifically, 10 mg of **Pt_1_Ag_28_-1** nanoclusters was dissolved in 30 mL of CH_2_Cl_2_. 300 μL of C_6_H_11_-*c*-SH was added and reacted for 2 hours at 40 °C. After the reaction was complete, the solution was rotavaporated, and then approximately 50 mL of methanol was added to wash the product. The precipitate was dissolved in CH_2_Cl_2_ giving rise to the solution of **Pt_1_Ag_28_-2** nanoclusters. The yield was about 80% (*i.e.*, 8 mg of **Pt_1_Ag_28_-2** was obtained).

### 2.4 Converting **Pt_1_Ag_28_-2** into **Pt_1_Ag_28_-1** nanoclusters

Specifically, 10 mg of **Pt_1_Ag_28_-2** nanoclusters was dissolved in 30 mL of CH_2_Cl_2_. 0.1 g of Adm-SH was added and reacted for 3 minutes at room temperature. After the reaction was complete, the solution was rotavaporated, and then approximately 50 mL of methanol was added to wash the product. The precipitate was dissolved in CH_2_Cl_2_ giving rise to the solution of **Pt_1_Ag_28_-1** nanoclusters. The yield was about 90% (*i.e.*, 9 mg of **Pt_1_Ag_28_-1** was obtained). Compared with the conversion from **Pt_1_Ag_28_-1** to **Pt_1_Ag_28_-2**, the reverse process is quicker.

### 2.5 Crystallization of the **Pt_1_Ag_28_-2** nanoclusters

Single crystals of **Pt_1_Ag_28_-2** were crystallized by vapor diffusion of ether into the CH_2_Cl_2_ solution of the nanoclusters over 7 days. For promoting the crystallization, Na^+^(BPh_4_)^–^ counter-ions (molar ratio between clusters and counter-ions was 1 : 2) were added into the CH_2_Cl_2_ solution. Then black crystals were collected and the structure of the **Pt_1_Ag_28_-2** nanocluster was determined. The CCDC number of the **Pt_1_Ag_28_-2** nanocluster is ; 1840953.

### 2.6 Test of the temperature–PL correlation

10 mg of **Pt_1_Ag_28_-1** (or **Pt_1_Ag_28_-2**) was dissolved in 10 mL of CH_2_Cl_2_/2-CH_3_-THF. Then the solutions were cooled to different temperatures and the PL spectra were measured.

### 2.7 Catalytic performance

For the preparation of catalysts, **Pt_1_Ag_28_-1** (or **Pt_1_Ag_28_-2**) clusters were supported on commercial carbon nanotubes (CNTs; Beijing Bo Yu high-tech new material technology Co., Ltd). The CNTs were first dispersed in toluene, and the nanoclusters were added to the suspension of CNTs under vigorous magnetic stirring. The adsorption of clusters was allowed to proceed overnight. Then the product was separated from the solution by centrifugation. The cluster@CNT composite was dried in a vacuum for 12 h; then, **Pt_1_Ag_28_-1**@CNT and **Pt_1_Ag_28_-2**@CNT catalysts (with a 2 wt% cluster loading) were obtained.

For the catalytic activity test, a 10 mL Schlenk bottle was charged with 0.5 mmol of styrene, 1.5 mmol of TBHP (*tert*-butyl hydroperoxide), 20 mg of cluster@CNT catalyst, 10 mg of K_2_CO_3_, and 2 mL of toluene. Then the suspension was stirred at 50 °C for 24 hours. The suspension was then centrifuged to remove solids, and the catalytic product was analysed by gas chromatography with an internal standard.

### 2.8 XAFS (X-ray absorption fine structure spectroscopy) measurements

XAFS measurements at the Pt L_3_-edge (11564 eV) were performed at the beamline BL14W1 station of the Shanghai Synchrotron Radiation Facility (SSRF), China. The storage ring of the SSRF was working at an energy of 3.5 GeV with an average electron current of 300 mA. The hard X-ray was monochromatized with a Si (311) monochromator. EXAFS data were collected in the transmission mode in the energy range from –200 below to 1000 eV above the Pt L_3_-edge. The acquired EXAFS data were processed according to the standard procedures using the ARTEMIS module implemented in the IFEFFIT software packages.

### 2.9 X-ray crystallography

The data collection for single crystal X-ray diffraction was carried out on a Bruker Smart APEX II CCD diffractometer under liquid nitrogen flow at 200 K, using graphite-monochromatized Mo Kα radiation (*λ* = 0.71073 Å). Data reductions and absorption corrections were performed using the SAINT and SADABS programs, respectively. The electron density was squeezed by Olex 2. The structure was solved by direct methods and refined with full-matrix least squares on *F*^2^ using the SHELXTL software package. All non-hydrogen atoms were refined anisotropically, and all the hydrogen atoms were set in geometrically calculated positions and refined isotropically using a riding model.

## 3 Theoretical methods

Density functional theory (DFT) calculations were employed to optimize the geometric structures and calculate the Kohn–Sham orbitals of Pt_1_Ag_28_ nanoclusters using the Perdew–Burke–Ernzerhof (PBE) GGA functional.^9^ The triple-zeta polarized (TZP) basis set with inclusion of the scalar relativistic effect *via* a zeroth-order regular approximation (ZORA) implemented in the ADF package was adopted.^10^ In the electronic structure analysis, the Kohn–Sham orbitals were calculated to analyze contributions of different atomic orbital types to molecular orbitals.

### 3.1 Characterization

UV-vis absorption spectra of nanoclusters dissolved in CH_2_Cl_2_ were recorded using an Agilent 8453 diode array spectrometer.

Electrospray ionization time-of-flight mass spectrometry (ESI-TOF-MS) measurement was performed using a MicrOTOF-QIII high-resolution mass spectrometer.

PL spectra were measured on an FL-4500 spectrofluorometer with the same optical density (OD) of ∼0.05. Of note, the PL excitation spectrum of **Pt_1_Ag_28_-1** or **Pt_1_Ag_28_-2** was measured at 600 or 750 nm, respectively, for suppressing the interference from each other (*e.g.*, **Pt_1_Ag_28_-1** fluoresces at 600 nm, while **Pt_1_Ag_28_-2** does not fluoresce at this wavelength).

Quantum yields (QYs) were measured with dilute solutions of nanoclusters on a HORIBA FluoroMax-4P.

Transmission electron microscopy (TEM) was conducted on a JEM-2100 microscope with an accelerating voltage of 200 kV.

Thermogravimetric analysis (TGA) was carried out on a thermogravimetric analyser (DTG-60H, Shimadzu Instruments, Inc.)

X-ray photoelectron spectroscopy (XPS) measurement was performed on a Thermo ESCALAB 250, configured with a monochromated Al Kα (1486.8 eV) 150 W X-ray source, 0.5 mm circular spot size, flood gun to counter charging effects, and analysis chamber base pressure lower than 1 × 10^–9^ mbar.

Inductively coupled plasma-atomic emission spectrometry (ICP-AES) measurements were performed on an Atomscan Advantage instrument made by Thermo Jarrell Ash Corporation.

Electrochemical measurements (differential pulse voltammetry, DPV) of clusters were performed with an electrochemical workstation (CHI 700E) using a Pt working electrode (0.4 mm diameter), a Pt wire counter electrode and an Ag wire quasi-reference electrode in 0.1 M Bu_4_NPF_6_–CH_2_Cl_2_. The electrolyte solution was deaerated with ultra-high purity nitrogen for 40 min and blanketed under a nitrogen atmosphere during the entire experimental procedure.

## 4 Results and discussion

### 4.1 Syntheses and crystallization

The **Pt_1_Ag_28_-1** nanoclusters were prepared using our previously reported procedure.8*b* The **Pt_1_Ag_28_-2** nanoclusters were synthesized by reacting pure **Pt_1_Ag_28_-1** with excess HS-*c*-C_6_H_11_ at 40 °C (see Experimental Methods for more details). After ∼2 hours, all the **Pt_1_Ag_28_-1** nanoclusters were completely converted into **Pt_1_Ag_28_-2** in a high yield (>80%, Ag atom basis). The as-prepared **Pt_1_Ag_28_-2** was crystallized by vapor diffusion of ether into a CH_2_Cl_2_ solution of the nanoclusters over 7 days. The **Pt_1_Ag_28_-1** structure was reported by us in early work.8*b* The structure of **Pt_1_Ag_28_-2** is newly determined in the current work. X-ray photoelectron spectroscopy (XPS) and inductively coupled plasma (ICP) measurements were performed to validate the ratio of Pt/Ag in the bi-metallic **Pt_1_Ag_28_-2** nanoclusters (Fig. S1, S2 and Table S1†), and the results perfectly matched the theoretical value (1/28 of Pt/Ag). Furthermore, the purity of **Pt_1_Ag_28_-2** was confirmed by thermogravimetric analysis (TGA). The experimental weight loss of 49.57% (Fig. S3†) is consistent with the calculated loss (49.26%) of the ligands (*i.e.*, PPh_3_ and S-*c*-C_6_H_11_) in **Pt_1_Ag_28_-2**. Both Pt_1_Ag_28_ nanoclusters are highly stable at 50 °C (Fig. S4;† clusters are dissolved in CHCl_3_, and in contact with air). In the DPV analysis (Fig. S5†), **Pt_1_Ag_28_-1** showed two oxidation peaks at 0.76 V (O1) and 1.28 V (O2) and a reduction peak at –1.32 V (R1); in comparison, **Pt_1_Ag_28_-2** exhibited three oxidation peaks at 0.75 V (O1), 1.08 V (O2) and 1.45 V (O3) and a reduction peak at –0.92 V (R1). The catalytic performance of both Pt_1_Ag_28_ nanoclusters in the oxidation of styrene was evaluated (Fig. S6,† and see the catalysis conditions in Experimental methods). The catalysis was performed at 50 °C to ensure that both clusters were stable. Although the catalytic selectivity for epoxide or benzaldehyde is similar in both catalysis, the overall catalytic conversion by **Pt_1_Ag_28_-2**@CNTs is much higher than that by **Pt_1_Ag_28_-1**@CNTs (66.48% *versus* 43.43%; shown in Fig. S6†). Such a difference may result from the ligand effect between the two nanoclusters.

**Fig. 1:**
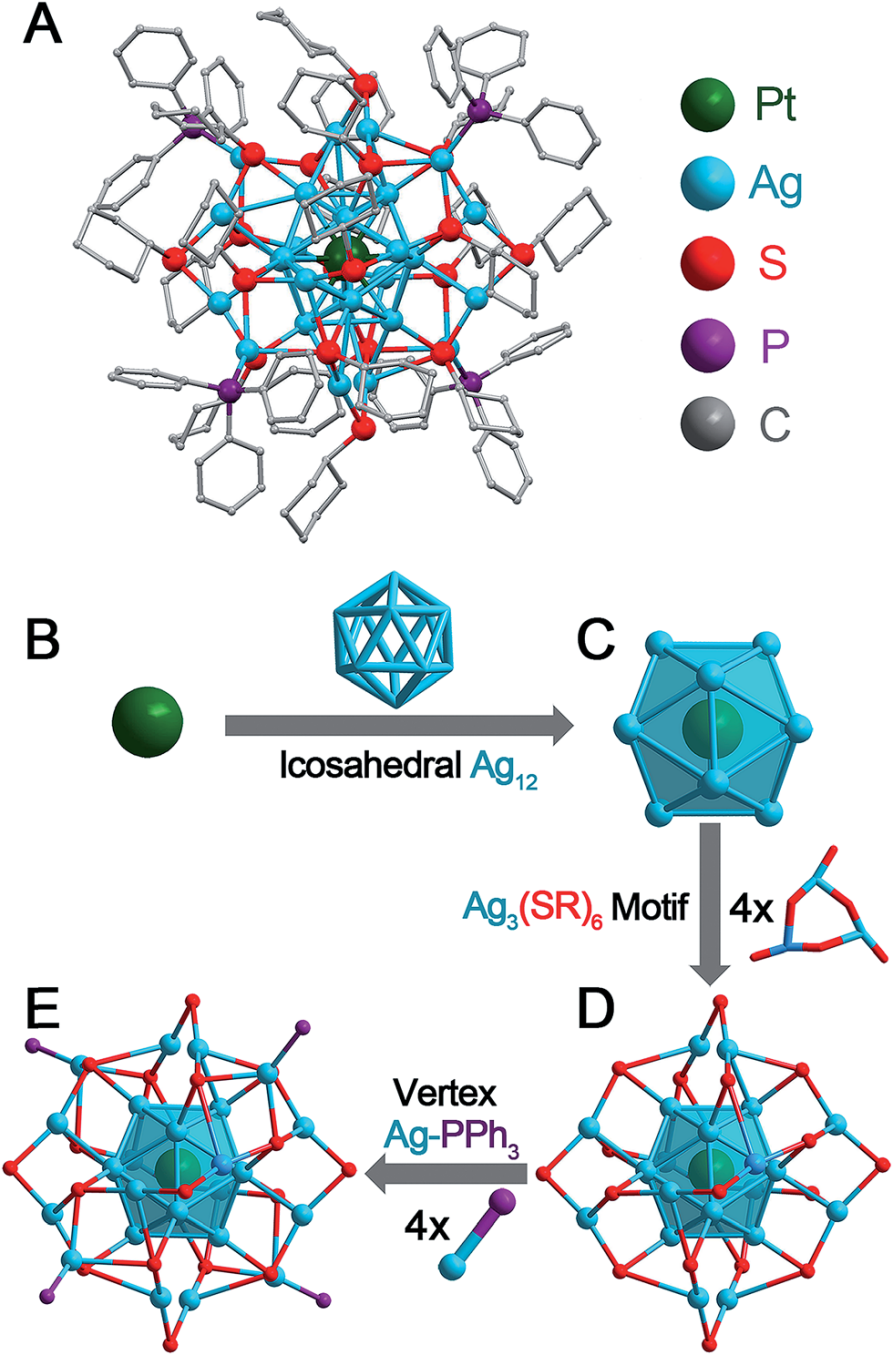
**Pt_1_Ag_28_-2** nanocluster and its structural anatomy. (A) Total structure. (B) Innermost Pt atom. (C) Pt_1_Ag_12_ kernel with an icosahedral configuration. (D) Pt_1_Ag_12_ kernel protected by four Ag_3_(S-Adm)_6_ motifs. These four motifs make up a large Ag_12_(S-Adm)_18_ motif wrapping the icosahedral kernel. (E) Four Ag-PPh_3_ architectures occupy the vertex positions of the nanocluster. Color codes: green sphere, Pt; blue spheres, Ag; red spheres, S; purple spheres, P; and grey spheres, C. For clarity, the hydrogen atoms are not shown.

The **Pt_1_Ag_28_-2** nanoclusters crystallize in the *P*2_1_/*c* space group. The structure of **Pt_1_Ag_28_-2** is shown in Fig. 1A and the structural anatomy is shown in Fig. 1B–E (see Fig. S7† for the complete structure). Specifically, a single Pt atom is arranged in the innermost position of the overall structure (Fig. 1B), which is further capped by an Ag_12_ cage, forming a Pt_1_Ag_12_ kernel with an icosahedral configuration (Fig. 1C). Furthermore, this Pt_1_Ag_12_ kernel is surrounded by four Ag_3_(S-*c*-C_6_H_11_)_6_ motifs. Of note, each Ag_3_(S-*c*-C_6_H_11_)_6_ motif links with three other motifs *via* sharing the terminal thiolates, which constitutes a cage-like Ag_12_(S-*c*-C_6_H_11_)_18_ motif structure fully surrounding the Pt_1_Ag_12_ kernel (Fig. 1D). Moreover, four Ag-PPh_3_ architectures occupy the four vacancies of the aforementioned Pt_1_Ag_12_@Ag_12_(S-*c*-C_6_H_11_)_18_ structure, giving rise to the final **Pt_1_Ag_28_-2** structure. It should be noted that the capped Ag_12_(SR)_18_@(Ag-PPh_3_)_4_ structure of **Pt_1_Ag_28_-2** is similar to that of **Pt_1_Ag_28_-1**.8*b* The TEM images of **Pt_1_Ag_28_-1** and **Pt_1_Ag_28_-2** indicate that the clusters are uniform in size of about 1.6 nm (Fig. S8†), and this agrees with that determined by X-ray structural analysis.

### 4.2 Reversible structure transformation

**Fig. 2:**
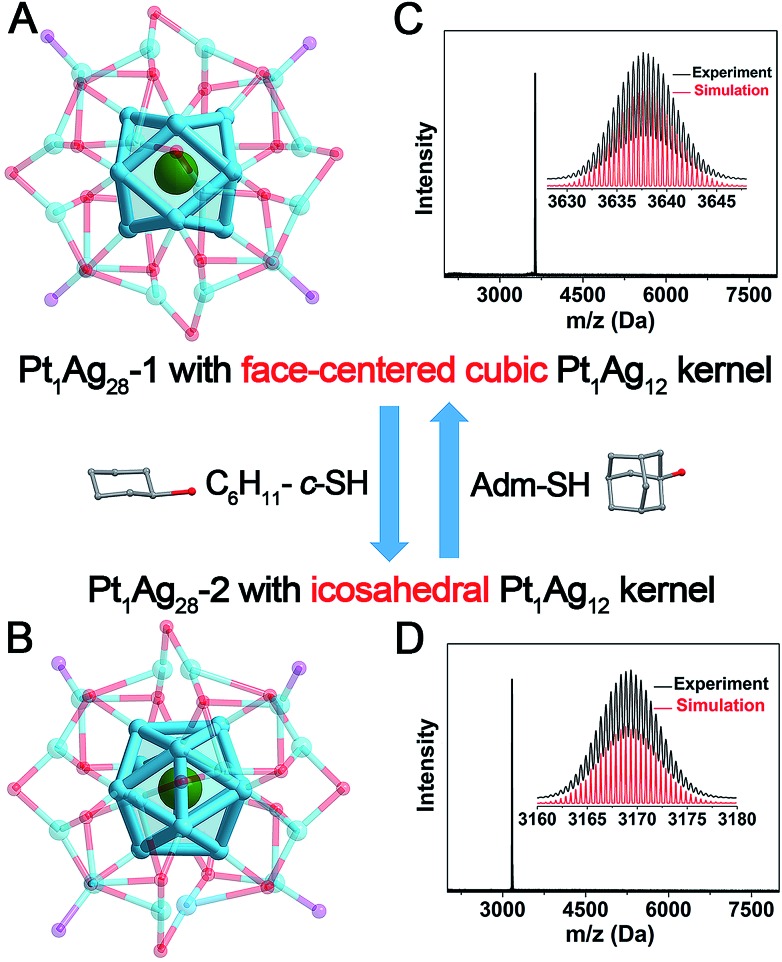
Illustration of the reversible transformation between (A) the **Pt_1_Ag_28_-1** nanocluster with a FCC Pt_1_Ag_12_ kernel and (B) the **Pt_1_Ag_28_-2** nanocluster with an icosahedral Pt_1_Ag_12_ kernel induced by the addition of the HS-*c*-C_6_H_11_ or HS-Adm ligand. ESI-MS spectra of (C) **Pt_1_Ag_28_-1** and (D) **Pt_1_Ag_28_-2** nanoclusters. Insets: experimental and simulated isotope patterns of each nanocluster. Color codes: green sphere, Pt; blue spheres, Ag; red spheres, S; and purple spheres, P. For clarity, the carbon and hydrogen atoms are not shown.

In order to elucidate the structural differences induced by the ligand exchange (*i.e.*, S-*c*-C_6_H_11_*versus* S-Adm), a comparison of the kernels of **Pt_1_Ag_28_-1** and **Pt_1_Ag_28_-2** is provided in Fig. 2. Both Pt_1_Ag_28_ nanoclusters comprise a Pt_1_Ag_12_ kernel; however, the FCC configuration of the Pt_1_Ag_12_ kernel in **Pt_1_Ag_28_-1** turns into the icosahedral configuration when the nanocluster converts to **Pt_1_Ag_28_-2**. The opposite process is also confirmed by reacting **Pt_1_Ag_28_-2** with excess HS-Adm. Previously, two nanoclusters following the isomerism phenomenon are almost arranged in the same molecular configuration, *e.g.*, Au_21_(S-Adm)_15_ and Au_21_(S-^*t*^Bu)_15_ isomers with a FCC configuration,^11^,^12^ Au_28_(S-Ph^*t*^Bu)_20_ and Au_28_(S-*c*-C_6_H_11_)_20_ isomers with a FCC configuration,6d,e Au_52_(S-Ph^*t*^Bu)_32_ and Au_52_(S-PhC_2_H_4_)_32_ isomers with a FCC configuration,6*c* Au_9_Ag_12_(S-Adm)_4_(dppm)_6_Cl_6_ and Au_9_Ag_12_(S-^*t*^Bu)_4_(dppm)_6_Cl_6_ with an icosahedral configuration,6*f* and so on.

Significantly, these two Pt_1_Ag_28_ isomers exhibit reversibility in terms of the kernel configuration between the FCC and icosahedron, which is observed for the first time in the nanocluster range (Fig. 2). Table 1 lists the comparison of bond lengths of Pt(core)–Ag(edge), Ag(edge)–Ag(edge), Ag(edge)–S(motif) and Ag(vertex)–P(vertex) between these two Pt_1_Ag_28_ isomers (see Fig. S9–S12† for the highlights of these types of bonds). Specifically, in **Pt_1_Ag_28_-1**, the bond lengths between the central Pt atom and the Ag atoms on the Pt_1_Ag_12_ shell range from 2.768 to 2.797 Å (average: 2.783 Å), whereas the Pt(core)–Ag(edge) distances in **Pt_1_Ag_28_-2** shorten to 2.744–2.791 Å (average: 2.763 Å). In contrast, the average Ag(edge)–Ag(edge) bond length of 2.801 Å in **Pt_1_Ag_28_-1** significantly increases to 2.915 Å (with a 4.07% difference) in **Pt_1_Ag_28_-2**. Furthermore, the average Ag(edge)–S bond length displays a 0.92% elongation in **Pt_1_Ag_28_-2** compared with that of the **Pt_1_Ag_28_-1** nanocluster. Moreover, the average Ag(vertex)–P bond length (2.400 Å) in **Pt_1_Ag_28_-2** is also slightly longer than that in **Pt_1_Ag_28_-1** (2.356 Å).

**Table 1:** Comparison of different bond lengths in **Pt_1_Ag_28_-1** and **Pt_1_Ag_28_-2** crystal structures

Bond length (Å)	**Pt_1_Ag_28_-1**	**Pt_1_Ag_28_-2**	Diff.
Pt(core)–Ag(edge)	2.768–2.797	2.744–2.791	0.72%
	Avg. 2.783	Avg. 2.763	
Ag(edge)–Ag(edge)	2.761–2.843	2.819–3.309	4.07%
	Avg. 2.801	Avg. 2.915	
Ag(edge)–S	2.438–2.498	2.480–2.517	0.92%
	Avg. 2.472	Avg. 2.495	
Ag(vertex)–P	2.292–2.384	2.395–2.409	1.87%
	Avg. 2.356	Avg. 2.400	

Electrospray ionization mass spectrometry (ESI-MS) was performed to verify the purity of each Pt_1_Ag_28_ isomer. As shown in Fig. 2C and D, the reaction between **Pt_1_Ag_28_-1** and HS-*c*-C_6_H_11_ decreases the mass value from 3637.64 Da to 3169.36 Da, which is assigned to **Pt_1_Ag_28_-2**. The magnification of the peak suggests a +2 charge state of **Pt_1_Ag_28_-2**, since this peak evidences a characteristic isotopic pattern with peaks separated by an *m*/*z* of 0.5 Da (in the positive mode). In this context, the overall charge state of these two Pt_1_Ag_28_ isomers is the same +2. Of note, the +2 charge state of **Pt_1_Ag_28_-2** matches the crystal data, because two negative (BPh_4_)^–^ counterions were observed in the crystal structure of **Pt_1_Ag_28_-2**. In addition, the gap between the mass peaks of two Pt_1_Ag_28_ isomers is calculated as 936.56 Da (*i.e.*, (3637.64 – 3169.36) × 2), which is in accordance with 18-fold the molecular weight gap between HS-Adm and HS-*c*-C_6_H_11_ ligands.

**Fig. 3:**
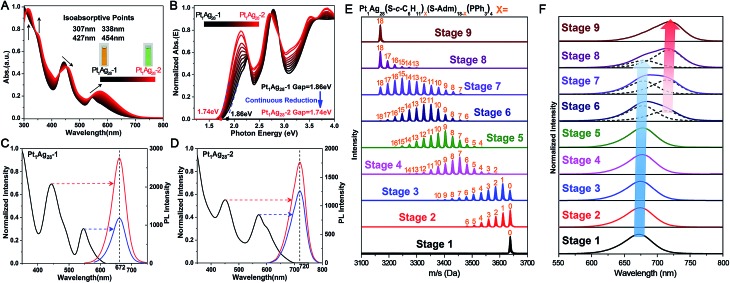
Real-time alternation of the (A) UV-vis absorption and (B) photon-energy plot from **Pt_1_Ag_28_-1** (in black) to **Pt_1_Ag_28_-2** (in red). Four isoabsorption points are observed at 307, 338, 427 and 454 nm, as depicted in (A). Insets of (A): photographs of the nanocluster solutions. Excitation spectrum (left) and emission spectra (right) of (C) **Pt_1_Ag_28_-1** and (D) **Pt_1_Ag_28_-2** at different excitation wavelengths, as indicated by the arrows. Time-dependent (E) ESI-MS spectra and (F) PL emission spectra from **Pt_1_Ag_28_-1** to **Pt_1_Ag_28_-2**. Insets of (F): dotted curves represent the separated overlapped curves. The blue arrow indicates the red-shift and reduction of the emission at ∼672 nm. The red arrow indicates the red-shift and enhancement of the emission at ∼720 nm.

It is accepted that the structures of nanoclusters play a decisive role in their chemical/physical properties.1a,b,1f–h The optical properties of Pt_1_Ag_28_ isomers are compared here to investigate the precise structure–property correlation. First of all, the time-dependent variation of UV-vis absorption from **Pt_1_Ag_28_-1** to **Pt_1_Ag_28_-2** was recorded. As shown in Fig. 3A, the absorptions at 545 and 443 nm in **Pt_1_Ag_28_-1** red-shift to 575 and 455 nm, respectively, in **Pt_1_Ag_28_-2**. Furthermore, the 333 and 305 nm absorption peaks in **Pt_1_Ag_28_-1** become more pronounced and red-shift to 350 and 315 nm, respectively. A total of four iso-absorption points are observed, centering at 307, 338, 427 and 454 nm. These iso-absorption points illustrate the high level of conversion from **Pt_1_Ag_28_-1** to **Pt_1_Ag_28_-2**. The solution of **Pt_1_Ag_28_-2** appears green to the naked eye, while the **Pt_1_Ag_28_-1** solution is orange (Fig. 3A, insets). The photon energy plots of the two Pt_1_Ag_28_ isomers were recorded (Fig. 3B), which demonstrate that the energy gap (between the highest occupied molecular orbital (HOMO) and the lowest unoccupied molecular orbital (LUMO)) of **Pt_1_Ag_28_-2** (1.74 eV) is much smaller than that of **Pt_1_Ag_28_-1** (1.86 eV); this is possibly due to the configuration transformation from the FCC to the icosahedron. In addition, accompanied by the ligand-exchange process from **Pt_1_Ag_28_-1** to **Pt_1_Ag_28_-2**, gradual reduction was detected in the HOMO–LUMO gap (Fig. 3B).

Fig. 3C and D show the PL performances of the Pt_1_Ag_28_ isomers. First of all, the PL excitation spectra are almost identical to the absorption spectra, which is reminiscent of the behavior of quantum-dots and some nanoclusters.8b,^13^ In addition, the emission peak wavelength is not dependent on the excitation wavelength but remains at 672 nm for **Pt_1_Ag_28_-1** and at 720 nm for **Pt_1_Ag_28_-2**. Furthermore, the emission of each isomer (*i.e.*, 1.84 eV of **Pt_1_Ag_28_-1** and 1.72 eV of **Pt_1_Ag_28_-2**) is very close to the HOMO–LUMO gap energy derived from the optical absorption spectrum. The extremely small difference in energy illustrates that the fluorescence corresponds to the HOMO–LUMO transition of each Pt_1_Ag_28_ isomer.

**Fig. 4:**
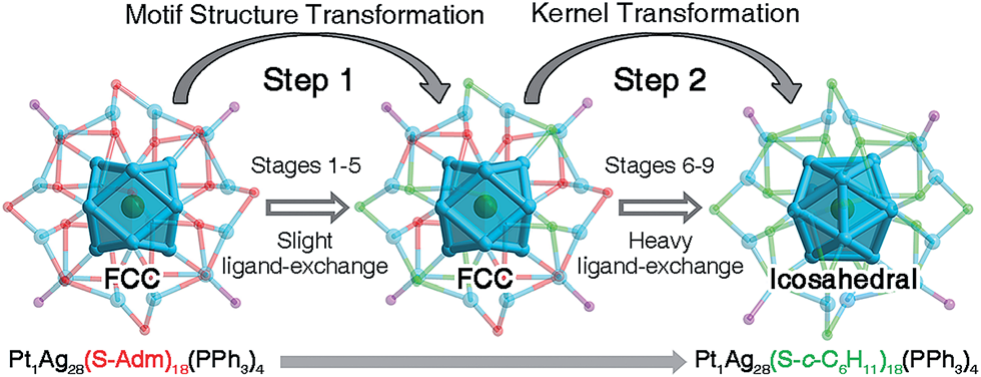
Proposed two-step transformation from **Pt_1_Ag_28_-1** to **Pt_1_Ag_28_-2**.

The structures of nanoclusters are determinant of their physical and chemical properties.1a,b,f–h In this context, the significant red-shift of the emission wavelength from **Pt_1_Ag_28_-1** to **Pt_1_Ag_28_-2** (∼50 nm) is traceable to the kernel transformation from the FCC to icosahedron. Considering that the kernel transformation is induced by the ligand exchange, we were motivated to track this configuration variation by matching the ESI-MS changes and the corresponding emission variations. Here, the ESI-MS and fluorescence variations from **Pt_1_Ag_28_-1** to **Pt_1_Ag_28_-2** are detected since the corresponding transformation is much slower than the reverse process, and thus is easier to monitor (see Fig. S13–S15† for the UV-vis, ESI-MS and PL variations from **Pt_1_Ag_28_-2** to **Pt_1_Ag_28_-1**). First of all, ESI-MS measurements were performed to monitor the ligand-exchange degree of each stage from **Pt_1_Ag_28_-1** to **Pt_1_Ag_28_-2** (Fig. 3E), and the time-dependent ligand-exchange process in mild and stepwise modes was deduced. Furthermore, the fluorescence variation can be divided into two periods (Fig. 3F, see the lateral comparison in Fig. S16†): (Period 1) from stage 1 to stage 5: each fluorescence curve only exhibits a single characteristic peak, although the emission red-shifts from 672 nm of stage 1 to 674, 675, 677, and 678 nm of stages 2–5, respectively; (Period 2) from stage 6 to stage 9: each emission curve contains two overlapping peaks, where the front peak decreases accompanying the ligand-exchange process, but the rear one increases gradually. Finally, the fluorescence curve just exhibits a single peak at 720 nm, which is the emission of pure **Pt_1_Ag_28_-2** (stage 9). Combining the variations of ESI-MS and fluorescence, the emission just displays a slight red-shift when the number of ligands exchanged on **Pt_1_Ag_28_-1** is less than 9–10 (stages 1–5, depicted by the blue arrow in Fig. 3F). When exchanging more ligands onto the nanocluster, a new peak emerges at 712 nm (stage 6). This new peak gradually red-shifts and increases to the final emission at 720 nm (stages 6–9, depicted by the red arrow in Fig. 3F), in which process the peak at ∼672 nm fades away. According to these phenomena, we propose that the transformation from **Pt_1_Ag_28_-1** to **Pt_1_Ag_28_-2** is a two-step process (Fig. 4): (i) the motif transformation process: the outmost motif structures alter gradually when inadequate S-*c*-C_6_H_11_ ligands are exchanged on the surface of the Pt_1_Ag_28_ nanocluster. In this process the FCC configuration of the Pt_1_Ag_12_ kernel remains (corresponding to stages 1–5); (ii) the kernel transformation process: sharp transformation of the kernel configuration from the FCC to icosahedron occurs as long as enough foreign ligands have been exchanged on the nanocluster surface, and finally all Pt_1_Ag_28_ nanoclusters contain the icosahedral Pt_1_Ag_12_ kernel (corresponding to stages 6–9). In addition, considering that the exchanged ligands do not come into contact with the innermost Pt_1_Ag_12_ kernel directly, we speculate that the kernel transformation occurs as a result of the motif transformation.

### 4.3 XAFS measurements

**Fig. 5:**
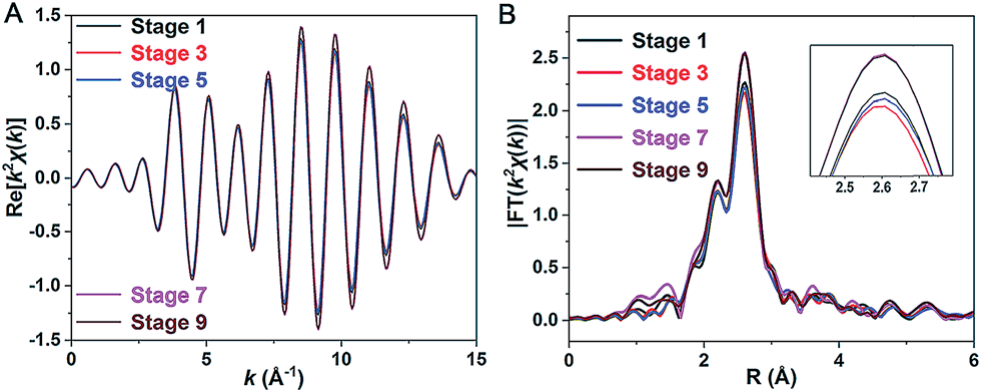
(A) *k*^2^*χ*(*k*) oscillations of Pt L_3_ and (B) corresponding Fourier transforms for Pt_1_Ag_28_ samples at different stages.

Our repeated attempts to crystallize the samples from stages 2–8 were unsuccessful, which might be because such Pt_1_Ag_28_ samples are protected by different proportions of S-Adm and S-*c*-C_6_H_11_ ligands (Fig. 3E, stages 2–8). XAFS measurements were performed for grasping the structural variation of Pt_1_Ag_28_ nanoclusters in different stages. XAFS results of stages 1, 3, 5, 7, and 9 are recorded since such results are capable of revealing the structural variations that correspond to the initial PL red-shift (672 nm → 678 nm) and the further abrupt changes (678 nm → 712 nm). As shown in Fig. 5 and Table 2 (see Fig. S17–S21† for more information), the fitted number of Pt–Ag bonds in each Pt_1_Ag_28_ sample is 12, which is in accordance with the Pt_1_Ag_12_ kernel in both FCC-Pt_1_Ag_28_ and icosahedral-Pt_1_Ag_28_ nanoclusters. Furthermore, the invariable bond lengths of Pt–Ag (remaining as 2.75 ± 0.01 Å, Table 2) in these samples validate the extremely small difference (cal. 0.72%, Table 1) in average Pt(core)–Ag(edge) bond lengths in **Pt_1_Ag_28_-1** and **Pt_1_Ag_28_-2**.

**Table 2:** Fitted EXAFS results of Pt_1_Ag_28_ samples corresponding to stages 1, 3, 5, 7, and 9 that relate to Fig. 3

Stage	Path	*N*	*R* (Å)	*σ* ^2^ (10^–3^ Å^2^)	Δ*E*_0_ (eV)
1	Pt–Ag	12	2.75 ± 0.01	6.9 ± 0.2	5.5 ± 0.6
3	Pt–Ag	12	2.75 ± 0.01	7.1 ± 0.2	5.3 ± 0.4
5	Pt–Ag	12	2.75 ± 0.01	7.0 ± 0.1	5.5 ± 0.4
7	Pt–Ag	12	2.75 ± 0.01	6.3 ± 0.3	5.7 ± 0.9
9	Pt–Ag	12	2.75 ± 0.01	6.3 ± 0.2	5.7 ± 0.7

Importantly, both the *k*^2^*χ*(*k*) oscillation and Fourier transform curves of stages 1–5 demonstrate that the Pt–Ag bonds are almost invariable in these stages (Fig. 5 and Table 2); however, the lower intensity of the Fourier transformed EXAFS *k*^2^*χ*(*k*) oscillations in stages 1–5 than in stages 7–9 indeed shows the higher disorder degree of Pt–Ag bonds in the former, *i.e.*, the larger Debye–Waller factor *σ*^2^ listed in Table 2. Generally, the Debye–Waller factor *σ*^2^ is a sum of two components, thermal disorder (*σ*_T_^2^) and structural disorder (*σ*_S_^2^), *i.e.*, *σ*^2^ = *σ*_T_^2^ + *σ*_S_^2^. From the single crystal XRD analysis (Table 1), the Pt–Ag bond lengths in **Pt_1_Ag_28_-1** span a narrower range of 0.029 Å (from 2.768 to 2.797 Å, average 2.783 Å) than that (0.047 Å, varying from 2.744 to 2.791 Å, average 2.763 Å) in **Pt_1_Ag_28_-2**, indicating a smaller structural disorder *σ*_S_^2^ in the FCC isomer. The larger *σ*^2^ but smaller *σ*_S_^2^ of the Pt–Ag bonds in **Pt_1_Ag_28_-1** than in icosahedral **Pt_1_Ag_28_-2** suggests that the thermal disorder *σ*_T_^2^ is larger in **Pt_1_Ag_28_-1**. It is known that when the measurement temperature *T* is close to or higher than the Einstein temperature of a bond, the thermal disorder *σ*_T_^2^ could be approximated by the correlated Einstein model: *σ*_T_^2^ = *k*_B_*T*/*k*_eff_, where *k*_B_ is the Einstein temperature and *k*_eff_ is the effective spring constant of the bond.^14^ Therefore, the larger thermal disorder *σ*_T_^2^ in **Pt_1_Ag_28_-1** indicates that the Pt–Ag interaction (reflected by the effective spring constant) is weaker in **Pt_1_Ag_28_-1** than in **Pt_1_Ag_28_-2**, or the Pt–Ag bond is strengthened in **Pt_1_Ag_28_-2**. This deduction is supported by the fact that, from the XRD analysis, the average Pt–Ag bond length (2.783 Å) in **Pt_1_Ag_28_-1** is longer by 0.02 Å than that (2.763 Å) in **Pt_1_Ag_28_-2**. More support could also be afforded by the Kohn–Sham molecular orbitals (MO) of both clusters yielded by DFT calculations (Fig. S22†). The bonding HOMO orbitals of **Pt_1_Ag_28_-1** are composed of 2.16% Pt 6sp, 2.29% Pt 5d, 26.03% Ag 5sp, 20.3% Ag 4d, and 41.67% S 3p orbitals, while the HOMO orbitals of **Pt_1_Ag_28_-2** are composed of 4.08% Pt 6sp, 1.24% Pt 5d, 39.79% Ag 5sp, 21.46% Ag 4d, and 26.97% S 3p orbitals. The smaller amount of Pt orbital components in **Pt_1_Ag_28_-1** than in **Pt_1_Ag_28_-2** (4.45% *versus* 5.22%) suggests the weaker Pt–Ag bonding in the former. This is also consistent with the consideration based on antibonding LUMO orbitals, where **Pt_1_Ag_28_-1** has a larger amount of Pt orbital components than **Pt_1_Ag_28_-2** (8.04% *versus* 7.33%).

EXAFS results indeed illustrate that the kernel transformation occurs within the ligand-exchange process from stage 5 to stage 7. Such a process (stage 5 → stage 7) is also regarded as the break point where significant PL variation occurs (Fig. 3F). In this context, by combining the ESI-MS, PL and EXAFS variation results, it has been unambiguously demonstrated that the nanocluster configuration transformation (from FCC to icosahedron) contains two discrete steps: the motif transformation and the kernel transformation. Specifically, in the early stage of the ligand-exchange process (stages 1–5), only the motif transformation occurs, which hardly affects the inner Pt–Ag bonds; when enough foreign ligands are exchanged on the Pt_1_Ag_28_ nanocluster (stages 5–9), remarkable transformation in the kernel occurs and the FCC configuration turns into the icosahedral configuration.

### 4.4 Temperature-dependent PL

**Fig. 6:**
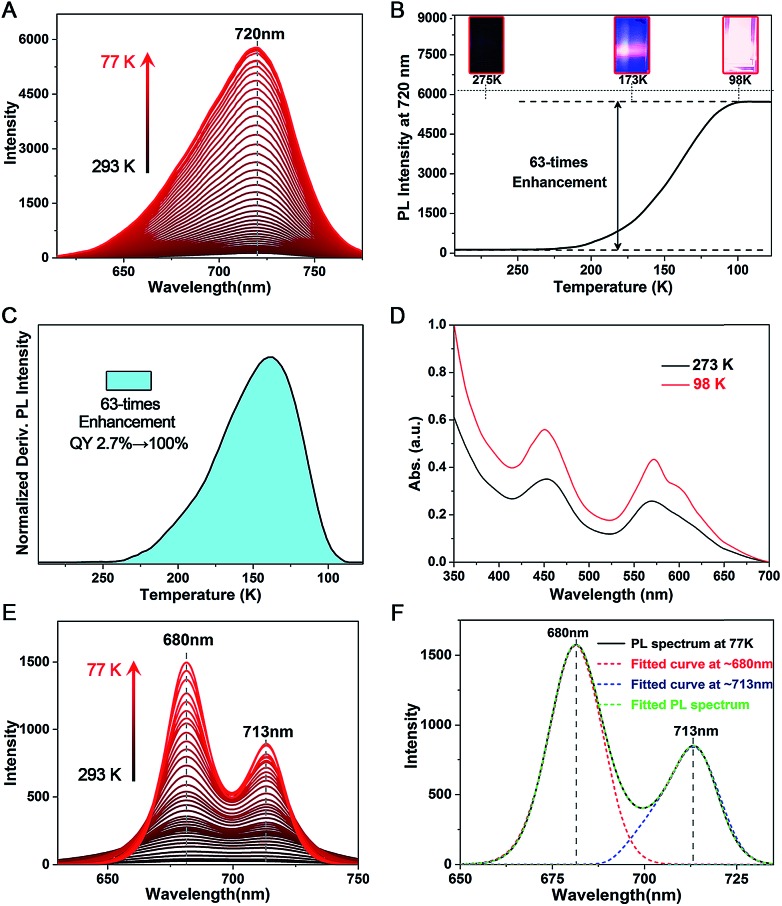
(A) PL variation of **Pt_1_Ag_28_-2** accompanying the reduction of temperature (from 293 K to 77 K, monitored per 3 K). (B) PL intensity at a fixed wavelength of 720 nm of **Pt_1_Ag_28_-2** at different temperatures. Insets: the digital photographs of **Pt_1_Ag_28_-2** solution under UV light. (C) Derivative result on the PL intensity of **Pt_1_Ag_28_-2**. (D) Temperature-dependent UV-vis absorption of **Pt_1_Ag_28_-2**. (E) PL variation of the intermediate ligand-exchange product (stage 7) accompanying the reduction of temperature. (F) Fitted curves of the PL spectrum of the intermediate ligand-exchange product (stage 7) measured at 77 K.

The PL QY of **Pt_1_Ag_28_-2** is only 2.7% at room temperature, which is much lower than that of **Pt_1_Ag_28_-1** (QY ∼ 4.9%). However, **Pt_1_Ag_28_-2** can emit bright-red light at low temperature (QY ∼ 100% at 98 K or lower temperature). Accordingly, the fluorescence intensity increased significantly (a 63-fold increase by comparing the 98 K data with the 293 K data) when the temperature is reduced to 98 K (Fig. 6A–C), and the UV-vis absorption presents a 1.8-fold enhancement (Fig. 6D). In this context, the PL QY of **Pt_1_Ag_28_-2** increases to almost 100%. In detail, when the temperature is higher than 250 K, the PL intensity is so weak that it cannot be observed by the naked eye (Fig. 6B, inset 275 K). When the temperature is continually reduced, the PL intensity enhances rapidly, and an obvious emission can be observed at 173 K (Fig. 6B, inset 173 K). Furthermore, the emission of **Pt_1_Ag_28_-2** nanoclusters is considerably bright when the temperature is reduced to 98 K or lower (Fig. 6B, inset 98 K). The enhanced PL intensity of **Pt_1_Ag_28_-2** is induced by the restrained thermal vibration (non-radiative transition) of the nanoclusters at low temperature, and thus the energy loss is just fluorescence (radiative transition).^15^ For comparison, the PL variation of **Pt_1_Ag_28_-1** accompanying the reduction of the temperature is exhibited in Fig. S23.†

Temperature-dependent PL spectra were also measured on the intermediate ligand-exchange Pt_1_Ag_28_ product. Specifically, we chose the product of stage 7 (see Fig. 3E and F) because the PL spectrum of this product displays the most obvious overlapping peaks. As shown in Fig. 6E, accompanying the reduction of temperature from 293 K to 77 K, the overlapped peaks become sharper and more separated. Meanwhile, the emission intensity appears to be significantly enhanced in the temperature reduction process. In this context, the peak-fitting becomes more easy and precise. Fig. 6F exhibits the overlapped curves from the PL spectrum of the intermediate ligand-exchange product (stage 7) measured at 77 K. Two independent curves were separated from the multi-peak PL spectrum, and they center at 680 and 713 nm, respectively. Of note, the fitting-peak positions are retained for these two separated curves; however, following the temperature reduction, the relative intensity of the fitted curve at ∼680 nm becomes stronger relative to the other one, which demonstrates the different emission performance of the FCC-Pt_1_Ag_28_ and icosahedral-Pt_1_Ag_28_ nanoclusters with the temperature reduction.

## 5 Conclusions

In summary, a ligand-exchange method was exploited to reversibly transform the **Pt_1_Ag_28_-1** nanoclusters with a FCC configuration and the **Pt_1_Ag_28_-2** nanoclusters with an icosahedral configuration. This is the first time that the isomerism phenomenon with reversible configurations has been observed. ESI-MS, PL and EXAFS results were combined to illustrate that the configuration transformation (between FCC and icosahedron) is a two-step process, including the outmost motif transformation process and the innermost kernel transformation process, where the latter transformation is induced by the former one. Based on these Pt_1_Ag_28_ isomers, the corresponding structure–optical property correlation was evaluated. UV-vis absorption, together with PL emission, demonstrates a reduced HOMO–LUMO gap of **Pt_1_Ag_28_-2** compared with that of **Pt_1_Ag_28_-1**. Overall, this work presents a nanocluster isomer system with a reversibly transforming configuration, which hopefully draws great attention of structural and theoretical chemists to fully understand the structural transformations as well as the structure–property correlation of metal nanoclusters.

## Conflicts of interest

There are no conflicts to declare.

## Supplementary Material

Supplementary information

Crystal structure data
