# Supplementary material for: Reversible nanocluster structure transformation between face-centered cubic and icosahedral isomers
Source: Chem Sci. 2019 Aug 5;10(37):8685–93. doi: 10.1039/c9sc02667c (PMC6849490; doi:10.1039/c9sc02667c)
Supplement: Supplementary file 1 [file SC-010-C9SC02667C-s001.pdf]

Electronic Supplementary Material (ESI) for Chemical Science.
This journal is © The Royal Society of Chemistry 2019

Supporting Information

Reversible nanocluster structure transformation between face-centered cubic and icosahedral isomers

Xi Kang,[a] Li Huang,[b] Wei Liu,[b] Lin Xiong,[c] Yong Pei,[c] Zhihu Sun,*[,b] Shuxin Wang,*[,a] Shiqiang Wei,[b] Manzhou Zhu*[,a]

[a]Department of Chemistry and Center for Atomic Engineering of Advanced Materials, Anhui Province Key Laboratory of Chemistry for Inorganic/Organic Hybrid Functionalized Materials, Anhui University, Hefei, Anhui 230601, China.

[b]National Synchrotron Radiation Laboratory, University of Science and Technology of China, Hefei, Anhui 230029, China.

[c]Department of Chemistry, Key Laboratory of Environmentally Friendly Chemistry and Applications of Ministry of Education, Xiangtan University, Xiangtan, Hunan 411105, China.

Corresponding Authors: zhsun@ustc.edu.cn (Z.S.); ixing@ahu.edu.cn (S.W.); zmz@ahu.edu.cn (M.Z.)

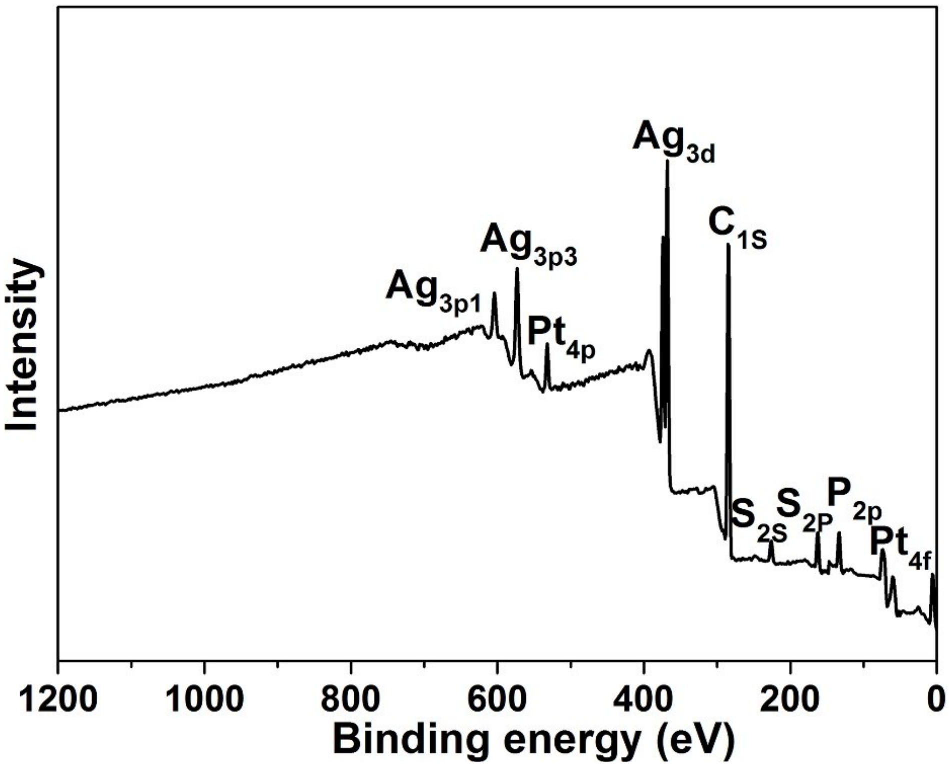

Fig. S1 XPS result of the $Pt_1Ag_{28}(S\text{-}c\text{-}C_6H_{11})_{18}(PPh_3)_4$ nanocluster.

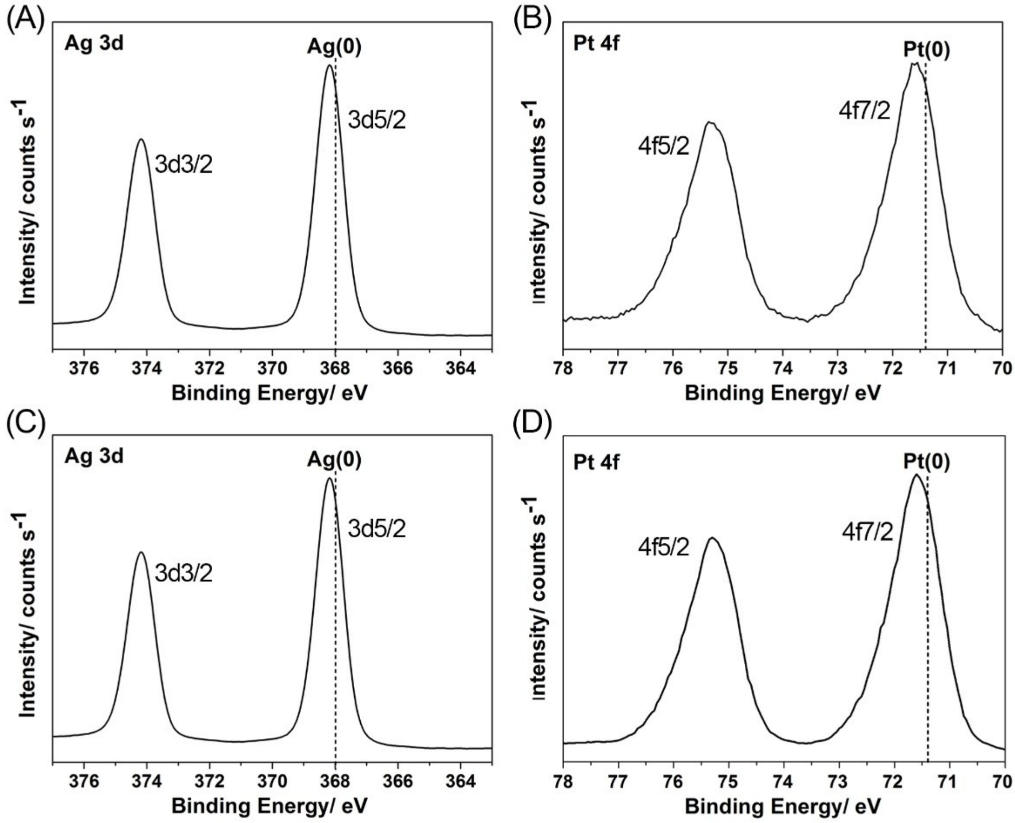

Fig. S2 XPS results of A) Pt 4f, B) Ag 3d of the $Pt_1Ag_{28}(S\text{-}c\text{-}C_6H_{11})_{18}(PPh_3)_4$ nanocluster, and C) Pt 4f, D) Ag 3d of the $Pt_1Ag_{28}(S\text{-}Adm)_{18}(PPh_3)_4$ nanocluster. The very similar XPS signals between them demonstrate that the valence states of metals are almost unchanged in the conversion process. Indeed, although the thiol ligands of Pt_1Ag_{28} cluster are exchanged in the conversion, both thiol are alkane ligands and the free electron numbers of both nanoclusters remain as 8, which can hardly affect the valence states of metals.

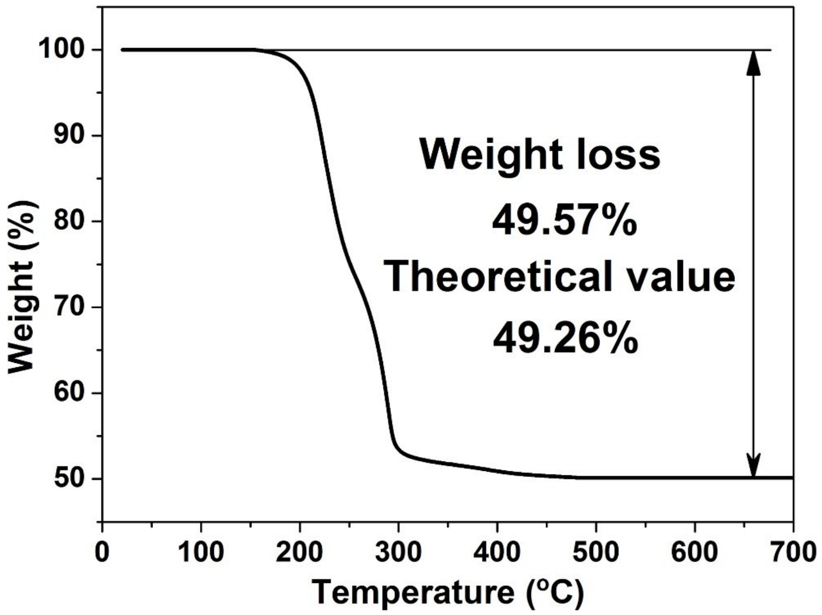

Fig. S3 TGA result of the $Pt_1Ag_{28}(S\text{-}c\text{-}C_6H_{11})_{18}(PPh_3)_4$ nanocluster.

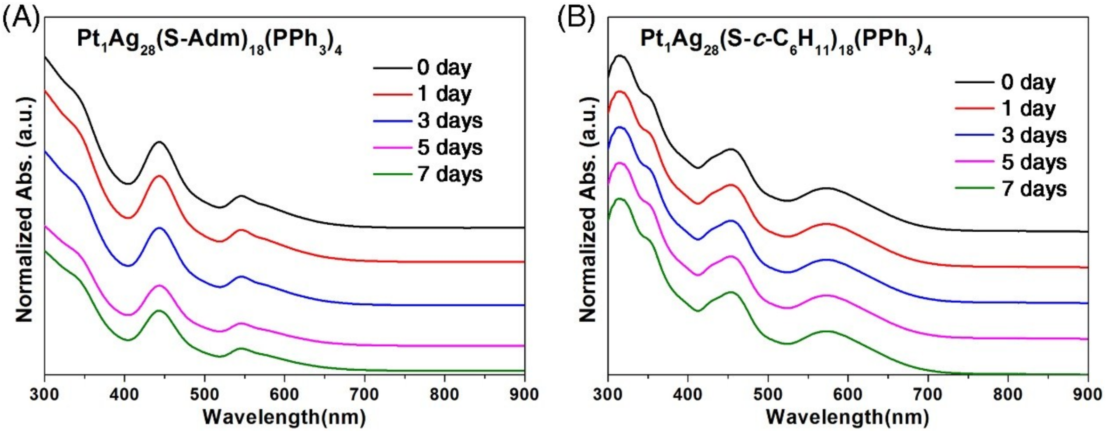

Fig. S4 Stability test of (A) $Pt_1Ag_{28}(S\text{-}Adm)_{18}(PPh_3)_4$ and (B) $Pt_1Ag_{28}(S\text{-}c\text{-}C_6H_{11})_{18}(PPh_3)_4$ nanoclusters at 50°C in contact with the air.

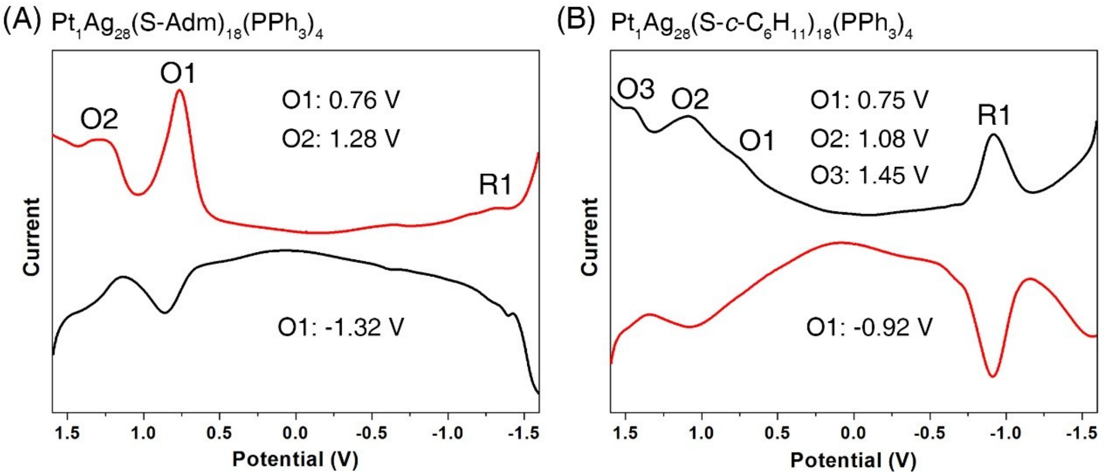

Fig. S5 DPV results of (A) $Pt_1Ag_{28}(S\text{-}Adm)_{18}(PPh_3)_4$ and (B) $Pt_1Ag_{28}(S\text{-}c\text{-}C_6H_{11})_{18}(PPh_3)_4$ nanoclusters.

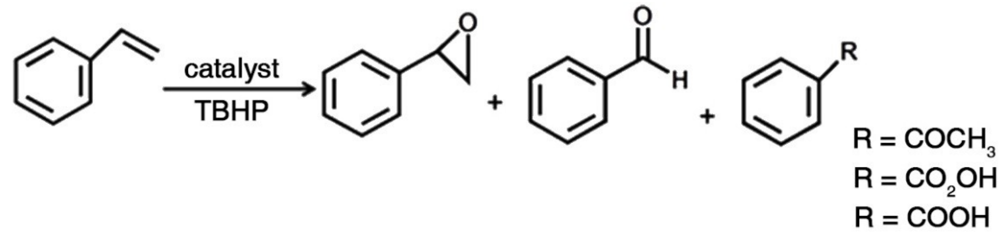

Entry[a]	Catalytic	Solvent	Conversion (%)[b]	Selectivity(%)[c]		
				Epoxide	Benzaldehyde	Other products
1	Pt_1Ag_{28}-1	Toluene	43.43	25.49	65.99	8.52
2	Pt_1Ag_{28}-2	Toluene	66.48	27.96	58.33	13.72

Fig. S6 The catalytic performance of both Pt_1Ag_{28} cluster catalyzers (Pt_1Ag_{28}-1: $Pt_1Ag_{28}(S\text{-}Adm)_{18}(PPh_3)_4$@CNTs; Pt_1Ag_{28}-2: $Pt_1Ag_{28}(S\text{-}c\text{-}C_6H_{11})_{18}(PPh_3)_4$@CNTs) in the oxidation of styrene. [a]Reaction conditions: 20 mg catalyst (2 wt% clusters loading), 57 mL (0.5 mmol) styrene, 144 μL (1.5 mmol) TBHP, 10 mg K_2CO_3, 2 mL toluene, 50 °C, 24 hours. [b]Conversion% = (converted styrene)/(initial amount of styrene) * 100%. [c]Determined by gas chromatography with internal standard.

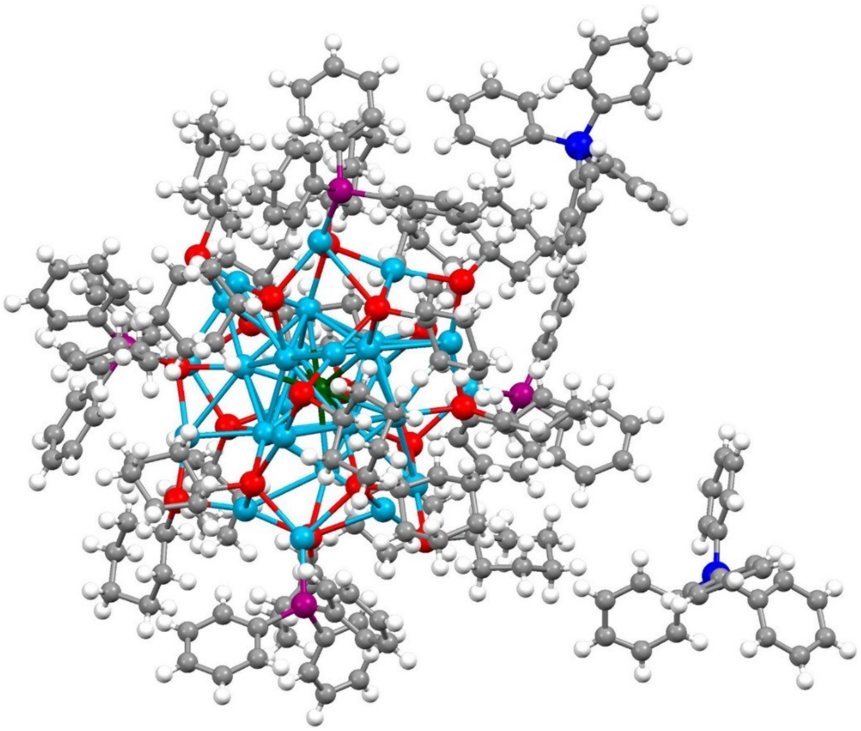

Fig. S7 Total structure of the $[Pt_1Ag_{28}(S\text{-}c\text{-}C_6H_{11})_{18}(PPh_3)_4](BPh_4)_2$ nanocluster. Color codes: green sphere, Pt; cerulean sphere, Ag; red sphere, S; purple sphere, P; blue sphere, B; grey sphere, C; white sphere, H.

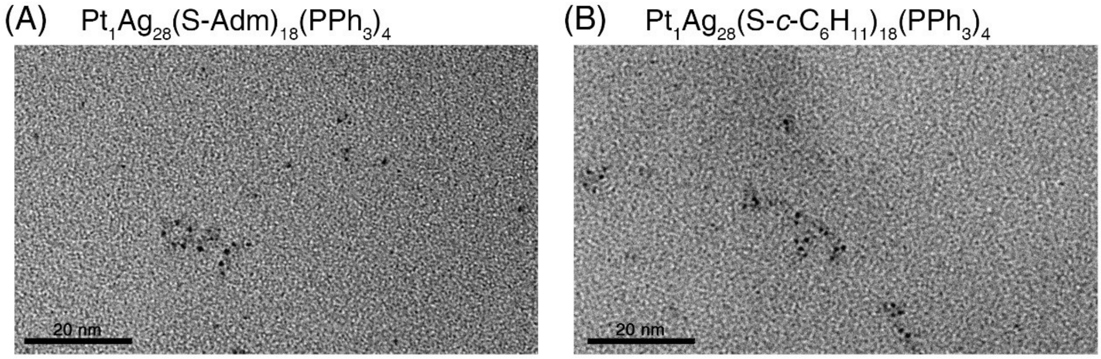

Fig. S8 TEM images of (A) $Pt_1Ag_{28}(S\text{-}Adm)_{18}(PPh_3)_4$ and (B) $Pt_1Ag_{28}(S\text{-}c\text{-}C_6H_{11})_{18}(PPh_3)_4$ nanoclusters. The clusters are uniform in size of about 1.6 nm, which agrees with that determined by x-ray structural analysis.

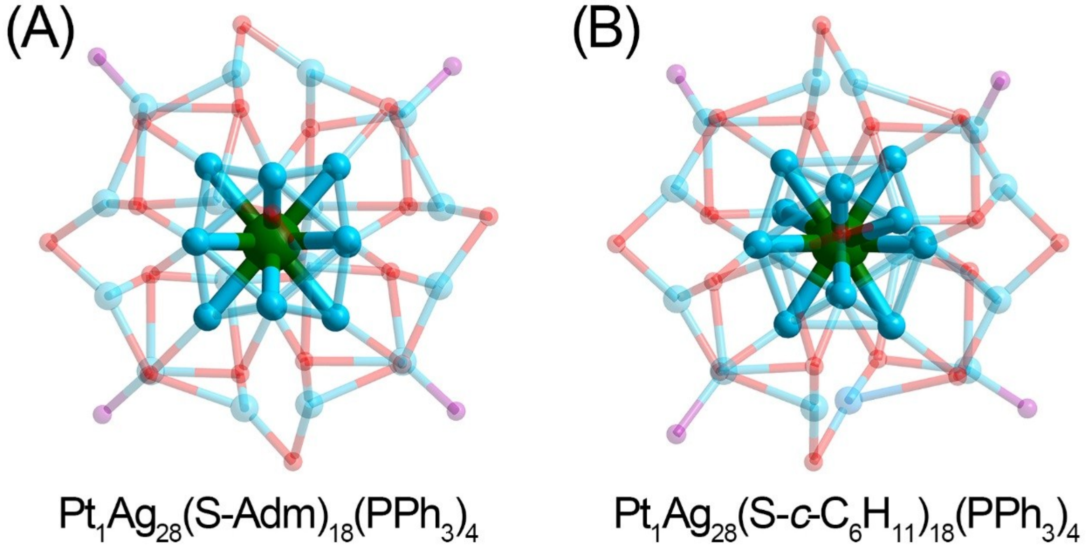

Fig. S9 Comparison of the Pt-Ag bond lengths (linking the Pt(core) and Ag(edge)) of $Pt_1Ag_{28}(S\text{-}Adm)_{18}(PPh_3)_4$ and $Pt_1Ag_{28}(S\text{-}c\text{-}C_6H_{11})_{18}(PPh_3)_4$ nanoclusters.

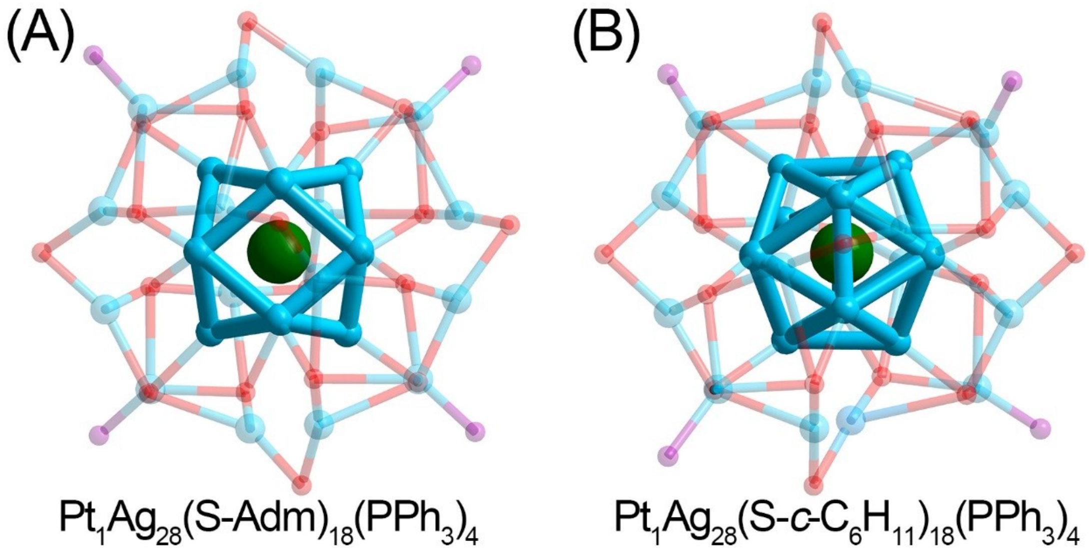

Fig. S10 Comparison of the Ag-Ag bond lengths (linking the Ag(edge) and Ag(edge)) of $Pt_1Ag_{28}(S\text{-}Adm)_{18}(PPh_3)_4$ and $Pt_1Ag_{28}(S\text{-}c\text{-}C_6H_{11})_{18}(PPh_3)_4$ nanoclusters.

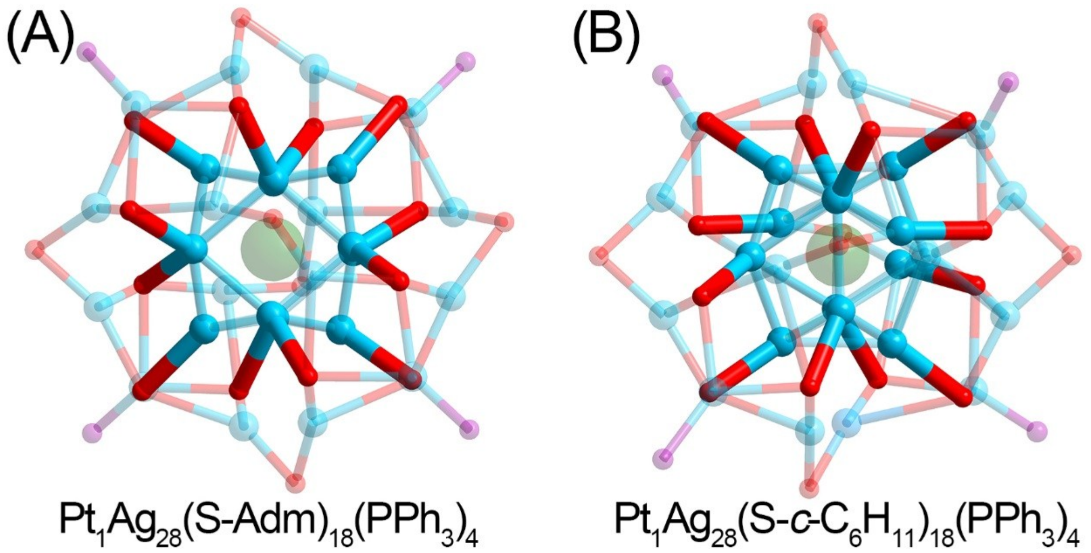

Fig. S11 Comparison of the Ag-S bond lengths (linking the Ag(edge) and S) of $Pt_1Ag_{28}(S\text{-}Adm)_{18}(PPh_3)_4$ and $Pt_1Ag_{28}(S\text{-}c\text{-}C_6H_{11})_{18}(PPh_3)_4$ nanoclusters.

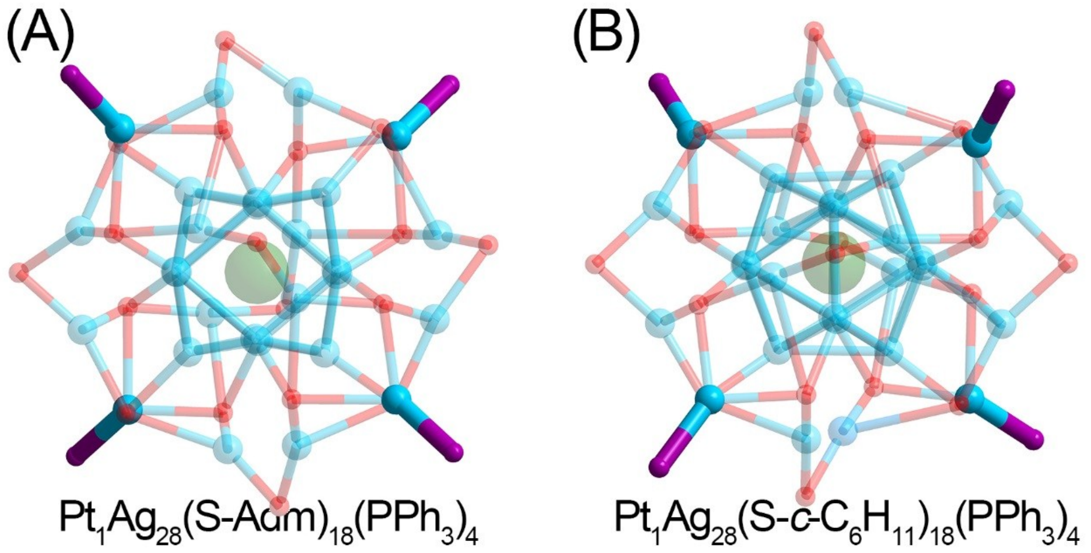

Fig. S12 Comparison of the Ag-P bond lengths (linking the Ag(vertex) and P) of $Pt_1Ag_{28}(S\text{-}Adm)_{18}(PPh_3)_4$ and $Pt_1Ag_{28}(S\text{-}c\text{-}C_6H_{11})_{18}(PPh_3)_4$ nanoclusters.

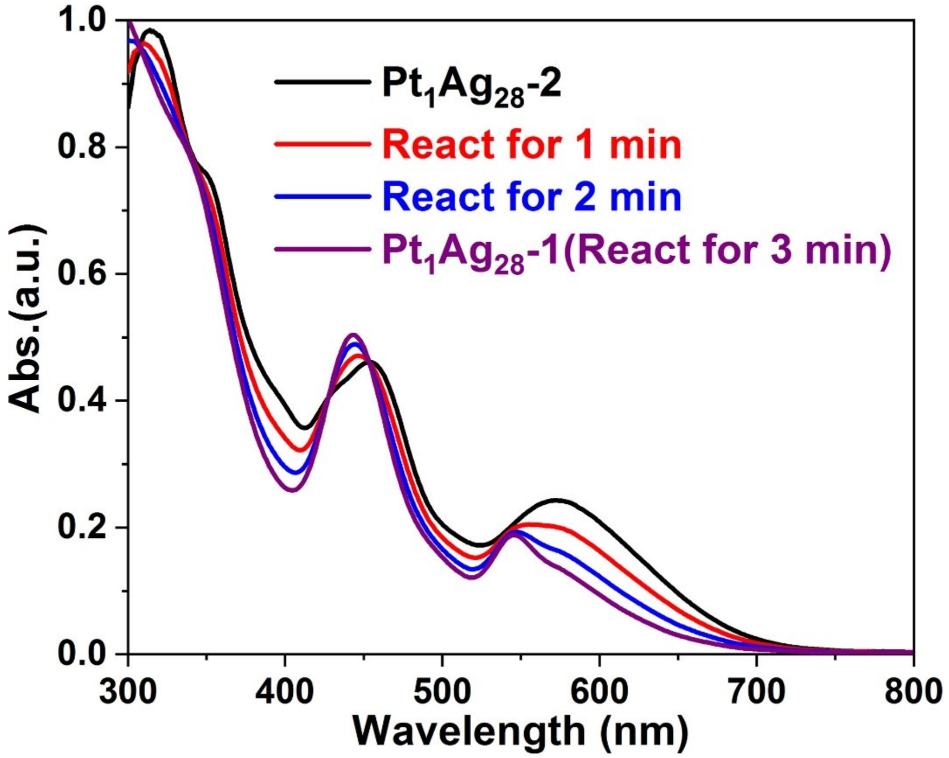

Fig. S13 Time-dependent UV-vis spectra from **Pt_1Ag_{28}-2** to **Pt_1Ag_{28}-1**.

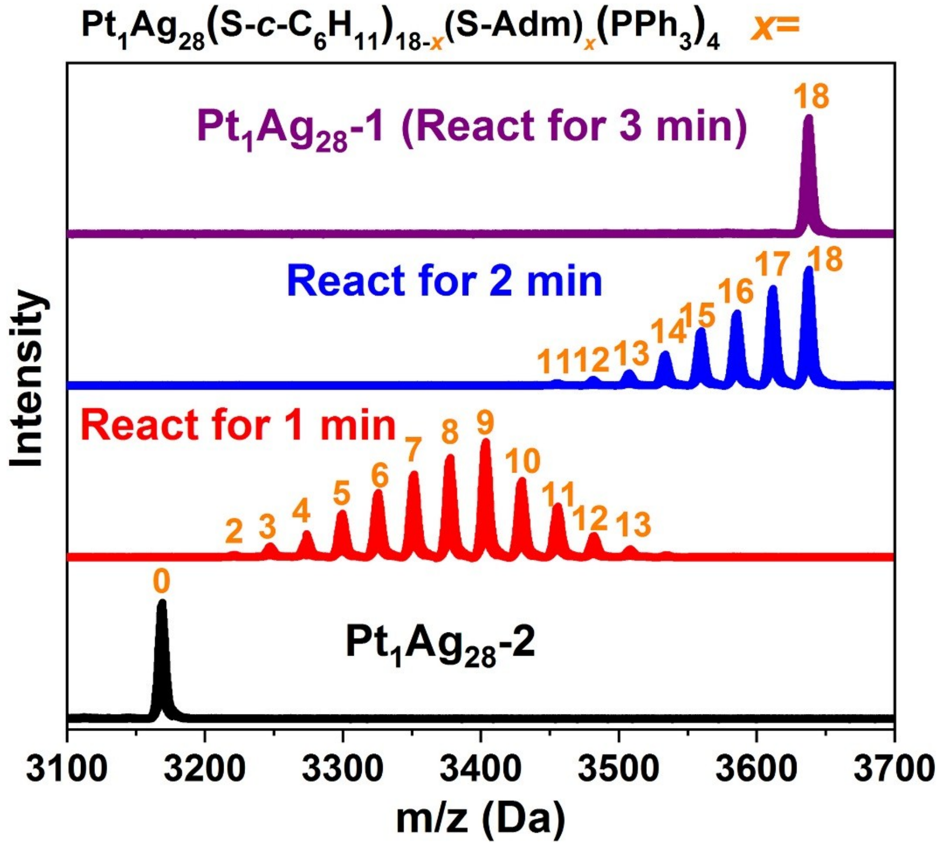

Fig. S14 Time-dependent ESI-MS results from **Pt_1Ag_{28}-2** to **Pt_1Ag_{28}-1**.

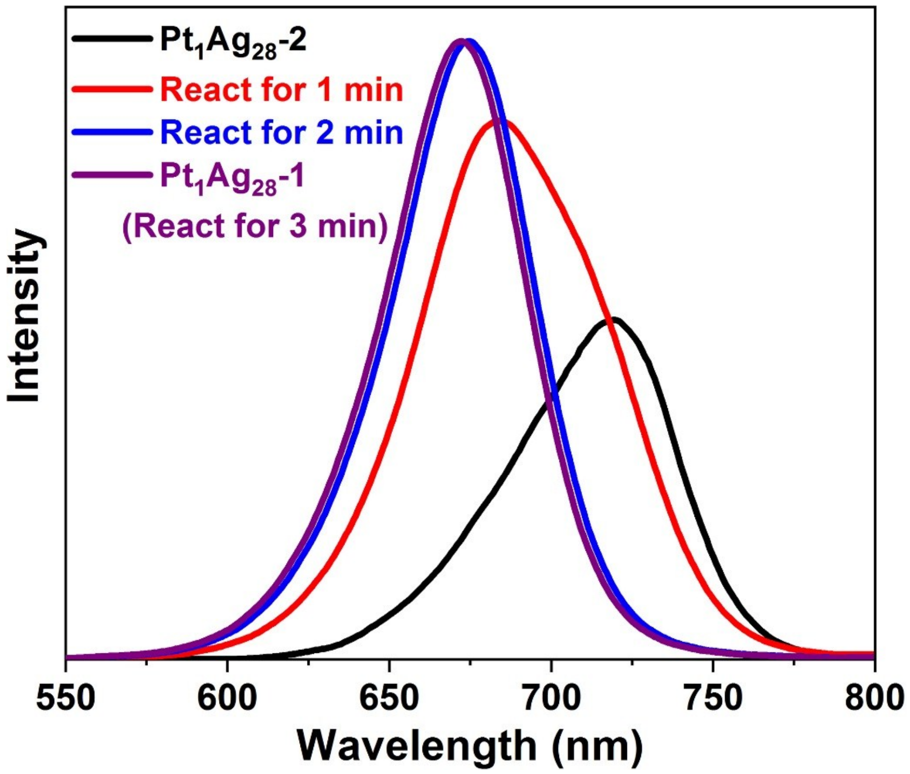

Fig. S15 Time-dependent PL emission spectra from **Pt_1Ag_{28}-2** to **Pt_1Ag_{28}-1**.

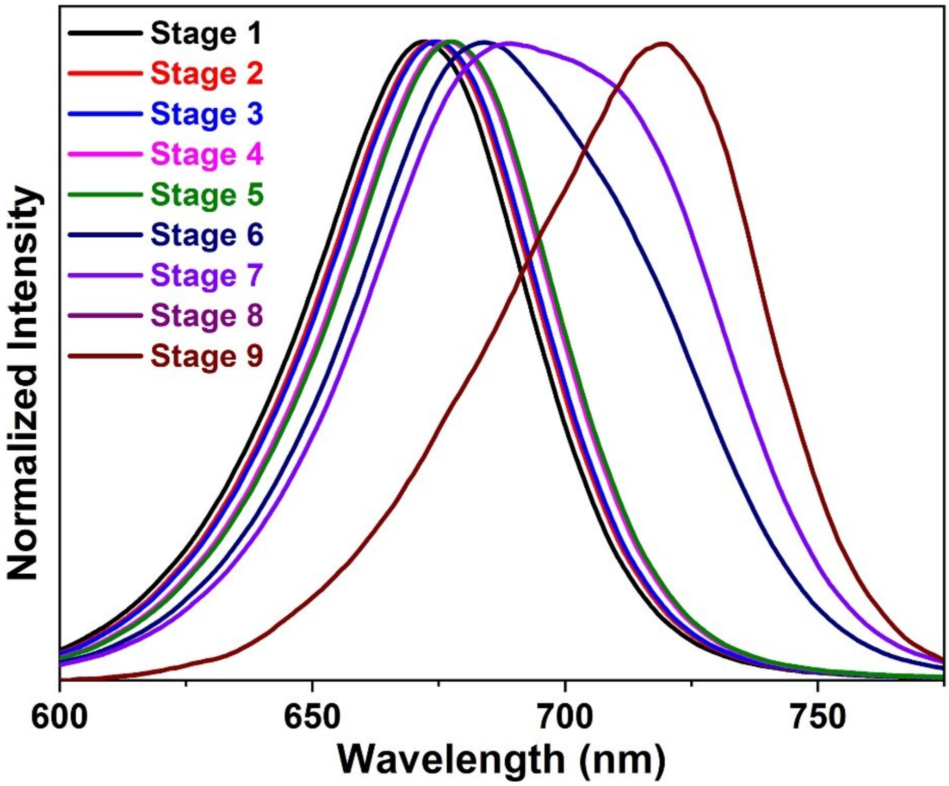

Fig. S16 Time-dependent PL emission spectra from **Pt_1Ag_{28}-1** to **Pt_1Ag_{28}-2**.

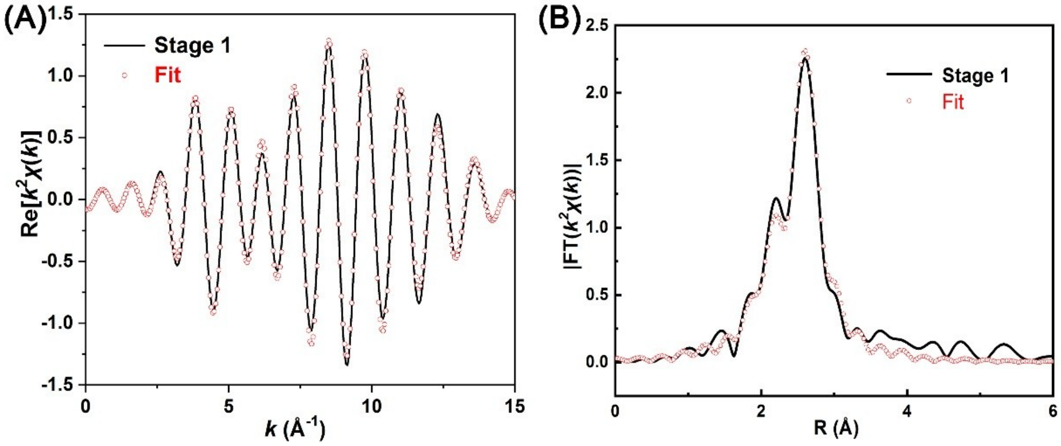

Fig. S17 (A) The fitting curve of $k^2\chi(k)$ oscillations of Pt L_3, and (B) corresponding Fourier transforms for Pt_1Ag_{28} sample from stage 1 corresponding to Figure 3.

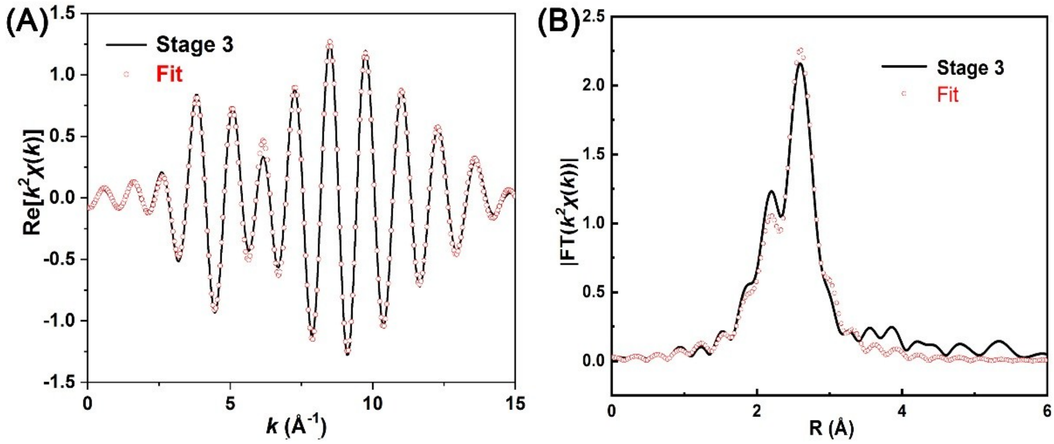

Fig. S18 (A) The fitting curve of $k^2\chi(k)$ oscillations of Pt L_3 and (B) corresponding Fourier transforms for Pt_1Ag_{28} sample from stage 3 corresponding to Figure 3.

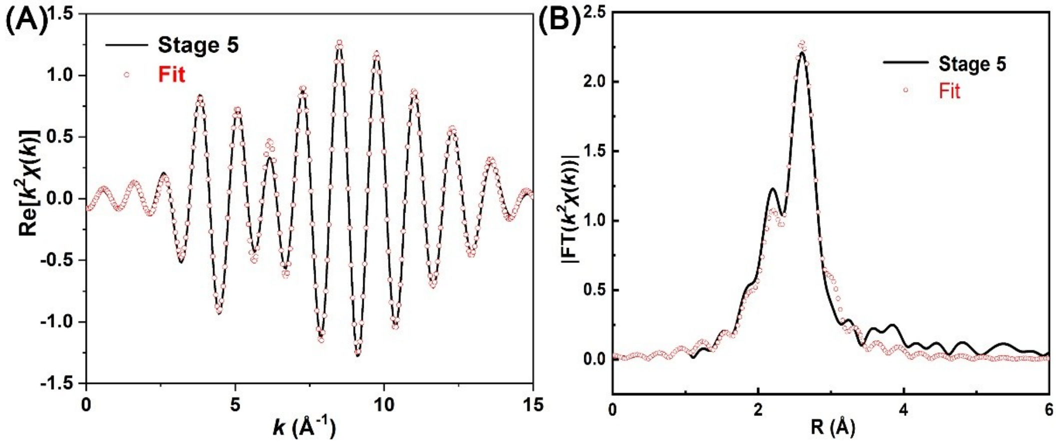

Fig. S19 (A) The fitting curve of $k^2\chi(k)$ oscillations of Pt L_3 and (B) corresponding Fourier transforms for Pt_1Ag_{28} sample from stage 5 corresponding to Figure 3.

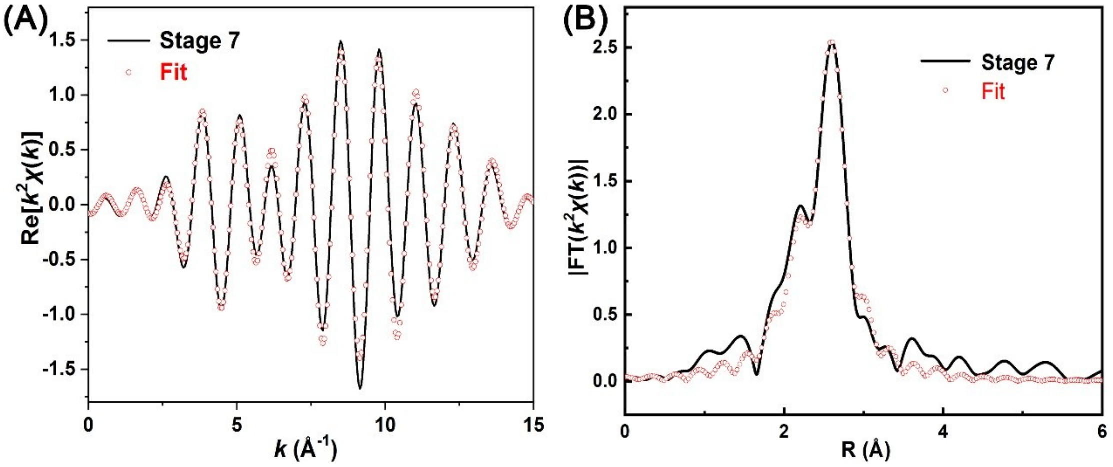

Fig. S20 (A) The fitting curve of $k^2\chi(k)$ oscillations of Pt L_3 and (B) corresponding Fourier transforms for Pt_1Ag_{28} sample from stage 7 corresponding to Figure 3.

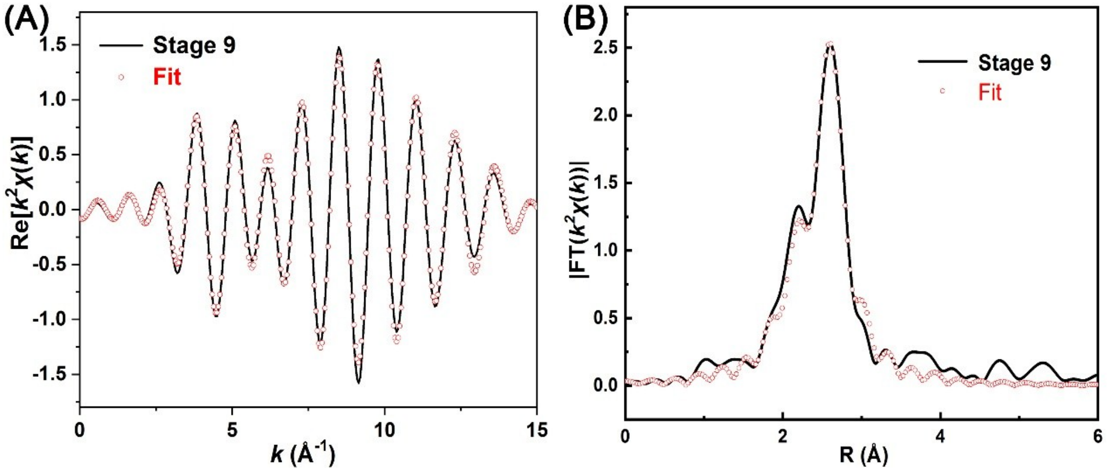

Fig. S21 (A) The fitting curve of $k^2\chi(k)$ oscillations of Pt L_3 and (B) corresponding Fourier transforms for Pt_1Ag_{28} sample from stage 9 corresponding to Figure 3.

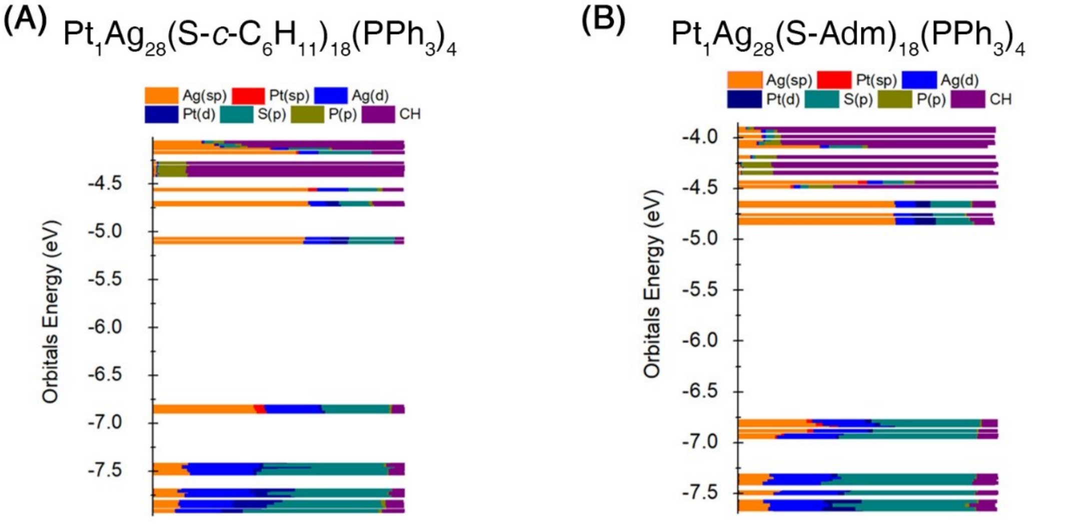

HOMO: Ag(sp) 39.79% Pt(sp) 4.08%
Ag(d) 21.46% Pt(d) 1.24% S(p) 26.97%
LUMO: Ag(sp) 59.43% Pt(sp) 0.02%
Ag(d) 10.38% Pt(d) 7.31% S(p) 18.26%

HOMO: Ag(sp) 26.03% Pt(sp) 2.16%
Ag(d) 20.3% Pt(d) 2.29% S(p) 41.67%
LUMO: Ag(sp) 60.08% Pt(sp) 0.06%
Ag(d) 7.33% Pt(d) 7.98% S(p) 14.25%

Fig. S22 Kohn-Sham molecular orbitals (MO) of (A) $Pt_1Ag_{28}(S\text{-}c\text{-}C_6H_{11})_{18}(PPh_3)_4$ and (B) $Pt_1Ag_{28}(S\text{-}Adm)_{18}(PPh_3)_4$.

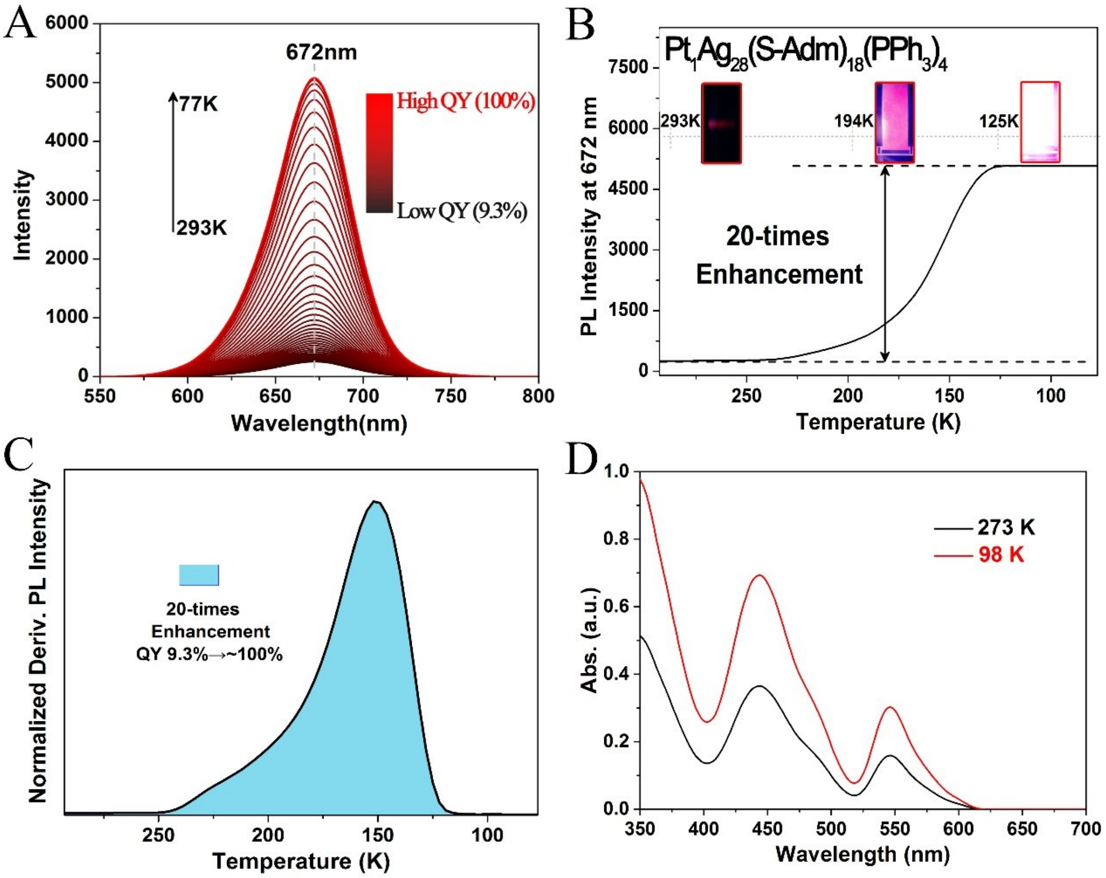

Fig. S23 (A) PL variation of the $Pt_1Ag_{28}(S\text{-}Adm)_{18}(PPh_3)_4$ nanocluster accompanying by the reduction of the temperature (from 293 K to 77 K, monitored per 3 K). (B) PL intensity on the fixed point of 672 nm of the $Pt_1Ag_{28}(S\text{-}Adm)_{18}(PPh_3)_4$ nanocluster under different temperatures. Insets: the digital photographs of the $Pt_1Ag_{28}(S\text{-}Adm)_{18}(PPh_3)_4$ nanocluster solution under the UV light. (C) Derivative result on the PL intensity of the $Pt_1Ag_{28}(S\text{-}Adm)_{18}(PPh_3)_4$ nanocluster. (D) Temperature-dependent UV-vis absorptions of the $Pt_1Ag_{28}(S\text{-}Adm)_{18}(PPh_3)_4$ nanocluster.

Table S1. Atom ratio of Pt/Ag calculated from ICP and XPS measurements of the Pt_1Ag_{28}(S-*c*-$C_6H_{11})_{18}(PPh_3)_4$ nanocluster.

	ICP experimental ratio	XPS experimental ratio	Theoretical ratio
Pt atom	4.15 %	3.60 %	3.44 %(1/29)
Ag atom	95.85 %	96.40 %	96.56 %(28/29)

Table S2. Crystal data and structure refinement for Pt_1Ag_{28}(S-*c*-$C_6H_{11})_{18}(PPh_3)_4$ nanocluster.

Identification code	Pt1Ag28
Empirical formula	C180 H256 Ag28 P4 Pt S18, 2(C24 H20 B), C4 H9 O
Formula weight	7048.78
Temperature/K	200(2)
Crystal system	monoclinic
Space group	P 1 21/c 1
a/Å	21.778(6)
b/Å	40.485(10)
c/Å	30.750(8)
α/°	90
β/°	99.764(3)
γ/°	90
Volume/Å^3	26720(12)
Z	4
ρ_{calc}g/cm^3	1.752
μ/mm^{-1}	2.728
F(000)	13832.0
Crystal size/mm^3	0.2 × 0.1 × 0.1
Radiation	MoKα (λ = 0.71073)
Index ranges	-25 ≤ h ≤ 25, -43 ≤ k ≤ 48, -36 ≤ l ≤ 36
Final R indexes [I>=2σ (I)]	R_1 = 0.1114, wR_2 = 0.2800
Final R indexes [all data]	R_1 = 0.1353, wR_2 = 0.2880
